# Temporal correlations between RBD-ACE2 blocking and binding antibodies to SARS-CoV-2 variants in CoronaVac-vaccinated individuals and their persistence in COVID-19 patients

**DOI:** 10.1038/s41598-025-98627-3

**Published:** 2025-05-06

**Authors:** Prapassorn Poolchanuan, Wasin Matsee, Adul Dulsuk, Rungnapa Phunpang, Chakkaphan Runcharoen, Thitiya Boonprakob, Onura Hemtong, Suchada Chowplijit, Vachara Chuapaknam, Tanaya Siripoon, Phimphan Pisutsan, Watcharapong Piyaphanee, Wathusiri Khongsiri, Nathamon Kosoltanapiwat, Le Van Tan, Susanna Dunachie, Chee Wah Tan, Lin-Fa Wang, Wasun Chantratita, Viravarn Luvira, Narisara Chantratita, SEACOVARIANTS, Prapassorn Poolchanuan, Adul Dulsuk, Le Van Tan, Chee Wah Tan, Narisara Chantratita, Nguyen To Anh, Nguyen Thi Thu Hong, Truong Hoang Chau Truc, Nguyen Thi Han Ny, Do Duong Kim Han, Le Kim Thanh, Lam Anh Nguyet, Cao Thu Thuy, Le Nguyen Truc Nhu, Tran Tan Thanh, Nguyen To Anh, Lam Minh Yen, Vu Thi Ty Hang, Pham Tieu Kieu, Vo Tan Hoang, Nguyen Thi Thao, Mary Chambers, Vu Duy Thanh, Tran Chieu Hoang, C. Louise Thwaites, Guy Thwaites, H. Rogier van Doorn, Trinh Son Tung, Guy Thwaites, Raph L. Hamers, Anuraj Shankar, Juthathip Mongkolsapaya, Gavin Screaton, Aiete Dijokaite-Guraliuc, Raksha Das, Chang Liu, Piyada Supasa, Muneeswaran Selvaraj, Susanna J. Dunachie, Paul Klenerman, E. Yvonne Jones, David I. Stuart, Barbara Kronsteiner-Dobramysl, Martha Zewdie, Priyanka Abraham, Jennifer Hill, Wang Lin-Fa, Wee Chee Yap, Beng Lee Lim, Suwarti, Yanie Tayipto, Eva Simarmata, Ragil Dien, Wanwisa Dejnirattisai, Warangkana Chantima, Apirak Junpirom, Vichapon Tiacharoen, Sophon Iamsirithaworn, Nicholas P.J. Day, Phaik Yeong Cheah, Tassawan Poomchaichote, Kanpong Boonthaworn, Nghiem My Ngoc, Alba Grifoni, Alessandro Sette

**Affiliations:** 1 Department of Microbiology and Immunology, Faculty of Tropical Medicine, Mahidol University, 420/6 Rajvithi Road, Bangkok, 10400 Thailand; 2 Department of Clinical Tropical Medicine, Faculty of Tropical Medicine, Mahidol University, Bangkok, Thailand; 3 Thai Travel Clinic, Hospital for Tropical Diseases, Faculty of Tropical Medicine, Mahidol University, Bangkok, Thailand; 4 Center for Medical Genomics, Faculty of Medicine Ramathibodi Hospital, Mahidol University, Bangkok, Thailand; 5 Faculty of Medical Technology, Huachiew Chalermprakiet University, Samut Prakan, Thailand; 6 Prachatipat Hospital, Thanya Buri, Pathum Thani Thailand; 7 Vichaivej International Hospital, Samut Sakhon, Thailand; 8 Oxford University Clinical Research Unit, Ho Chi Minh City, Vietnam; 9 Centre for Tropical Medicine and Global Health University of Oxford, Oxford, UK; 10 NDM Centre for Global Health Research, Nuffield Department of Clinical Medicine, University of Oxford, Oxford, UK; 11 Mahidol-Oxford Tropical Medicine Research Unit, Faculty of Tropical Medicine, Mahidol University, Bangkok, Thailand; 12 Programme in Emerging Infectious Diseases, Duke-NUS Medical School, Singapore, Singapore; 13 Infectious Diseases Translational Research Programme, Department of Microbiology and Immunology, Yong Loo Lin School of Medicine, National University of Singapore, Singapore, Singapore; 14 Vaccine Trial Centre, Faculty of Tropical Medicine, Mahidol University, Bangkok, Thailand; 15 Oxford University Clinical Research Unit, Ha Noi, Vietnam; 16 University of Oxford, Oxford, UK; 17 Oxford University Clinical Research Unit, Jakarta, Indonesia; 18 Faculty of Medicine, Siriraj Hospital, Mahidol University, Bangkok, Thailand; 19 Ministry of Public Health, Nonthaburi, Thailand; 20 Hospital for Tropical Diseases, Ho Chi Minh City, Vietnam; 21 La Jolla Institute for Immunology, La Jolla, CA USA

**Keywords:** SARS-CoV-2, COVID-19, RBD-ACE2 blocking antibody, Anti-spike antibody, Correlation, Vaccine, Immunology, Microbiology

## Abstract

Antibodies play a crucial role in protection against SARS-CoV-2. Understanding the correlation between binding and functional antibodies is essential to determine whether binding antibody levels can reliably predict neutralizing activity. We assessed antibody responses in 111 individuals vaccinated with the inactivated vaccine CoronaVac and 111 COVID-19 patients in Thailand. Plasma levels of ACE2-blocking antibodies targeting the receptor-binding domain (RBD) of SARS-Co-V2 variants were measured before vaccination and at 14 and 28 days after the second dose using a multiplex surrogate virus neutralization test. Anti-spike and anti-nucleocapsid antibodies were quantified by electrochemiluminescence immunoassay, and anti-RBD IgG by ELISA. After vaccination, blocking, anti-spike, and IgG antibody levels increased but declined rapidly within a month, whereas antibody levels in COVID-19 patients increased and persisted. Blocking and anti-spike antibody correlated at day 14 post-vaccination but not at day 28. In COVID-19 patients, correlations were moderate at day 14, and stronger at day 28. Correlations were weaker for Omicron subvariants than for the ancestral strain and non-Omicron variants. The weak correlation between blocking and anti-RBD IgG suggests binding antibodies might not predict neutralizing activity. These findings highlight the temporal nature of CoronaVac-induced immunity and the need for booster doses and variant-adapted vaccine.

## Introduction

The global pandemic caused by severe acute respiratory syndrome coronavirus 2 (SARS-CoV-2) has led to unprecedented public health challenges since its emergence in late 2019^1^. The rapid spread and evolution of the virus have resulted in the continuous emergence of new variants, complicating infection control and vaccination efforts. The SARS-CoV-2 receptor-binding domain (RBD) of the spike protein, has undergone numerous mutations throughout the pandemic, contributing to the emergence of variants such as Omicron and its subvariants, which have significantly influenced viral transmissibility, virulence, and immune escape mechanisms^2–4^. The emergence of the JN.1 subvariant as the predominant strain globally^4,5^, along with the recent increase in the prevalence of the KP.2 variant, underscores the ongoing evolution of SARS-CoV-2 and its potential impact on future outbreaks^6^.

Thailand, like many other countries, has experienced waves of different SARS-CoV-2 strains, including the Alpha, Beta, Delta, and Omicron variants^7,8^. These variants presented varying challenges in terms of transmission rates, disease severity, and vaccine effectiveness, requiring continuous monitoring and adaptation of public health strategies^7^. Understanding the antibody-based immune response to different variants is critical for developing effective public health interventions.

Neutralizing antibodies (nAbs) play a crucial role in the immune response against SARS-CoV-2 by blocking viral entry through targeting the spike RBD. The assessment of antibody responses involves measuring the levels of nAbs and binding antibodies, which provide insights into the immune protection and vaccine efficacy^9–11^. Previous studies have examined these antibodies in COVID-19 patients and vaccinated individuals, revealing variations in their responses to different viral variants^12–15^. Notably, emerging subvariants such as XBB.1.5, BA.2.86, and JN.1 have been associated with marked reductions in nAbs levels in both mRNA-vaccinated individuals and convalescent patients compared with the ancestral strain^16,17^. These findings underscore the need for continuous monitoring of vaccine induced immunity as new variants emerge.

In Southeast Asia, initial vaccination efforts primarily utilized the inactivated vaccine such as CoronaVac, followed by a mixture of viral vector, mRNA, and subunit vaccines^18–21^. This differs from Western countries, where viral vector and mRNA vaccines were predominantly used^22,23^. Given these differences, it is essential to understand the correlation between nAb and binding antibody levels in various contexts. Several studies demonstrated a strong correlation between SARS-CoV-2 anti-spike protein antibody levels as measured by electrochemiluminescence assay (ECLIA) and nAbs levels as detected by a neutralization assay in COVID-19 patients and mRNA vaccine recipients^24–27^. These findings suggest that ECLIA may be a reliable method for quantifying the antibody response, essential for estimating immune protection^24,28^.

A previous study reported a strong correlation between nAb levels measured using a neutralization detection kit (cPass, GenScript, USA) and anti-RBD IgG antibody levels measured using an IgG detection kit in CoronaVac-vaccinated individuals or those who received the ChAdOx1-S vaccine following CoronaVac vaccination^29^. However, such correlations have not been extensively evaluated in CoronaVac-vaccinated individuals, and limited information exists on the longitudinal changes in the correlations as immune responses develop over time.

To address these gaps, this study utilized the surrogate virus neutralization test (sVNT), an ELISA-based assay that detects functional antibodies capable of blocking the SARS-CoV-2 spike RBD-ACE2 interaction. sVNT offers distinct advantages, including the ability to perform without live virus, enabling its use under Biosafety Level-2 (BSL-2) conditions. Additionally, sVNT exhibits minimal cross-reactivity with other human coronaviruses while maintaining high specificity for SARS-CoV-2. Previous studies have demonstrated a strong correlation between sVNT and the gold-standard plaque reduction neutralization Test (PRNT), validating its use as a practical and scalable alternative for assessing functional antibodies against SARS-CoV-2^30–34^.

This study analyzed the levels of blocking antibodies detected by sVNT and binding antibodies and their correlation in individuals vaccinated with CoronaVac and COVID-19 patients in Thailand. As part of the broader SEACOVARIANTS consortium project focusing on the antibody-based immune response to SARS-CoV-2 variants in Southeast Asian populations, we enrolled 111 CoronaVac-vaccinated individuals and 111 COVID-19 patients in Thailand. We assessed blocking antibody levels using multiplex beads coated with RBD proteins from the ancestral strain and 12 variants multiplex sVNT, which have demonstrated strong correlation with live virus neutralization^30–34^. Anti-spike antibody levels were measured using ECLIA^20,24^, and anti-RBD IgG antibody levels were determined using in-house enzyme-linked immunosorbent assay (ELISA)^35,36^. Plasma samples from both cohorts were analyzed on days 0, 14, and 28 post-enrollment.

## Results

### Demographics and clinical characteristics of vaccinated individuals and COVID-19 patients

**Fig. 1:**
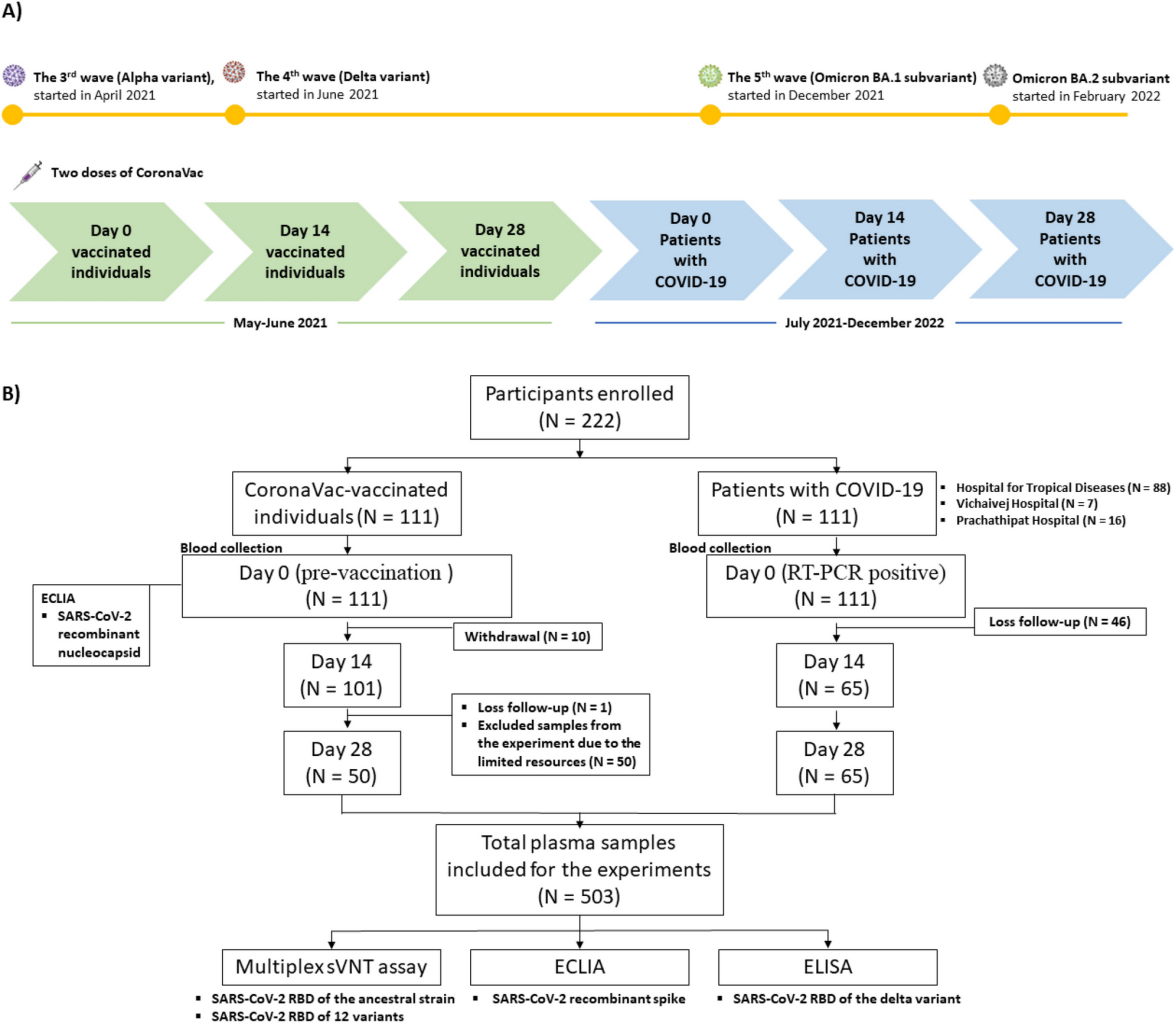
Timeline of sample collections and dominant circulating variants during the study period (**A**) and study flow diagram (**B**). This prospective study enrolled 111 CoronaVac-vaccinated individuals from May to June 2021^20^ and 111 COVID-19 patients from three hospitals in central Thailand from July 2021 to December 2022^30,35^. Plasma samples were collected on days 0, 14, and 28. RBD-ACE2 blocking antibody levels against the SARS-CoV-2 ancestral strain and 12 variants were assessed using multiplex sVNT^30^. Anti-spike antibody levels were measured using ECLIA, and anti-RBD IgG antibodies specific to the SARS-CoV-2 RBD of the Delta variant were determined by ELISA^35^.

In total, 222 participants were enrolled in this study and our previous studies, including 111 CoronaVac-vaccinated individuals^20^ and 111 COVID-19 patients^30,35^. Delta and Omicron were the dominant circulating variants during our study period, as shown in the timeline of sample collections and the dominant circulating variants (Fig. 1). The demographics and characteristics of the participants are presented in Table 1. The median age of vaccinated individuals was 35 years [interquartile range (IQR), 28–47 years], and 76 of 111 participants (68.5%) were women. All vaccinated individuals received two doses of the CoronaVac vaccine 21–28 days apart, and 63.3% had no underlying diseases. Meanwhile, 32 individuals had pre-existing conditions including hypertension (6, 5.4%), diabetes mellitus (5, 4.5%), dyslipidemia (8, 7.2%), and obesity with BMI > 30 kg/m^2^ (12, 10.8%). The number of participants decreased from 111 on day 0 to 101 and 100 on days 14 and 28, respectively, because of withdrawal and loss to follow-up.

**Table 1:** Demographics and clinical characteristics of CoronaVac-vaccinated individuals and COVID-19 patients.

Characteristics	Vaccinated individuals^a^ (N = 111)	COVID-19 patients^b^ (N = 111)
Demographics		
Age (years), median (IQR)	35 (28–47)	53 (41–63)
Male, n (%)	35 (31.5%)	45 (40.5)
Female, n (%)	76 (68.5%)	66 (59.5)
Vaccination history, n (%)		
Unvaccinated	0	17 (15.3)
Homologous inactivated vaccine (1–2 doses)	111 (100)	10 (10.6)
Homologous viral vector vaccine (1–2 doses)	0	31 (33.0)
Homologous mRNA vaccine (2–3 doses)	0	4 (4.3)
Heterologous vaccine (2–5 doses)***	0	49 (52.1)
Pre-existing conditions, n (%)		
No underlying disease	79 (63.3%)	47 (42.3)
Hypertension	6 (5.4%)	35 (31.5)
Dyslipidemia	8 (7.2%)	30 (27.0)
Diabetes mellitus	5 (4.5%)	18 (16.2)
Chronic heart disease	0	8 (7.2)
Obesity (BMI > 30)	12 (10.8%)	20 (18.0)
Asthma	3 (2.7%)	4 (3.6)
Cancer	0	1 (0.9)
Rheumatologic disease	0	5 (4.5)
Day to enrollment, median (IQR)	ND	1 (0–2)
Pneumonia	ND	41 (36.9)
Organ involved and complications, n (%)		
Lung	ND	64 (57.7)
Liver	ND	13 (11.7)
Kidney	ND	5 (4.5)
In-hospital death, n (%)	ND	0

^a^Data of 30 participants were obtained from^20^.

^b^Data of 111 participants were obtained from^30,35^.

The median age of COVID-19 patients was 53 years (IQR, 41–63 years), and 66 patients (59.5%) were women. Among the patients, 17 (15.3%) were unvaccinated before SARS-CoV-2 infection, and 94 patients with COVID-19 (84.7%) received 1–5 vaccine doses (median, 2 doses; IQR, 2–4 doses) prior to infection. Within this group, 10.6% received homologous inactivated vaccine, 33% received homologous viral vector vaccine, 4.3% received homologous mRNA vaccine, whereas 52.1% received heterologous vaccines. Among all patients, 64 (57.7%) had comorbidities, most commonly hypertension (35, 31.5%), dyslipidemia (30, 27.0%), diabetes mellitus (18, 16.2%), and obesity (20, 18%). Sixty-four patients (57.7%) experienced lung involvement and complications, and 41 (36.9%) developed pneumonia. All patients survived to hospital discharge.

### RBD-ACE2 blocking, anti-spike, and anti-RBD IgG antibody levels in CoronaVac-vaccinated individuals

**Fig. 2:**
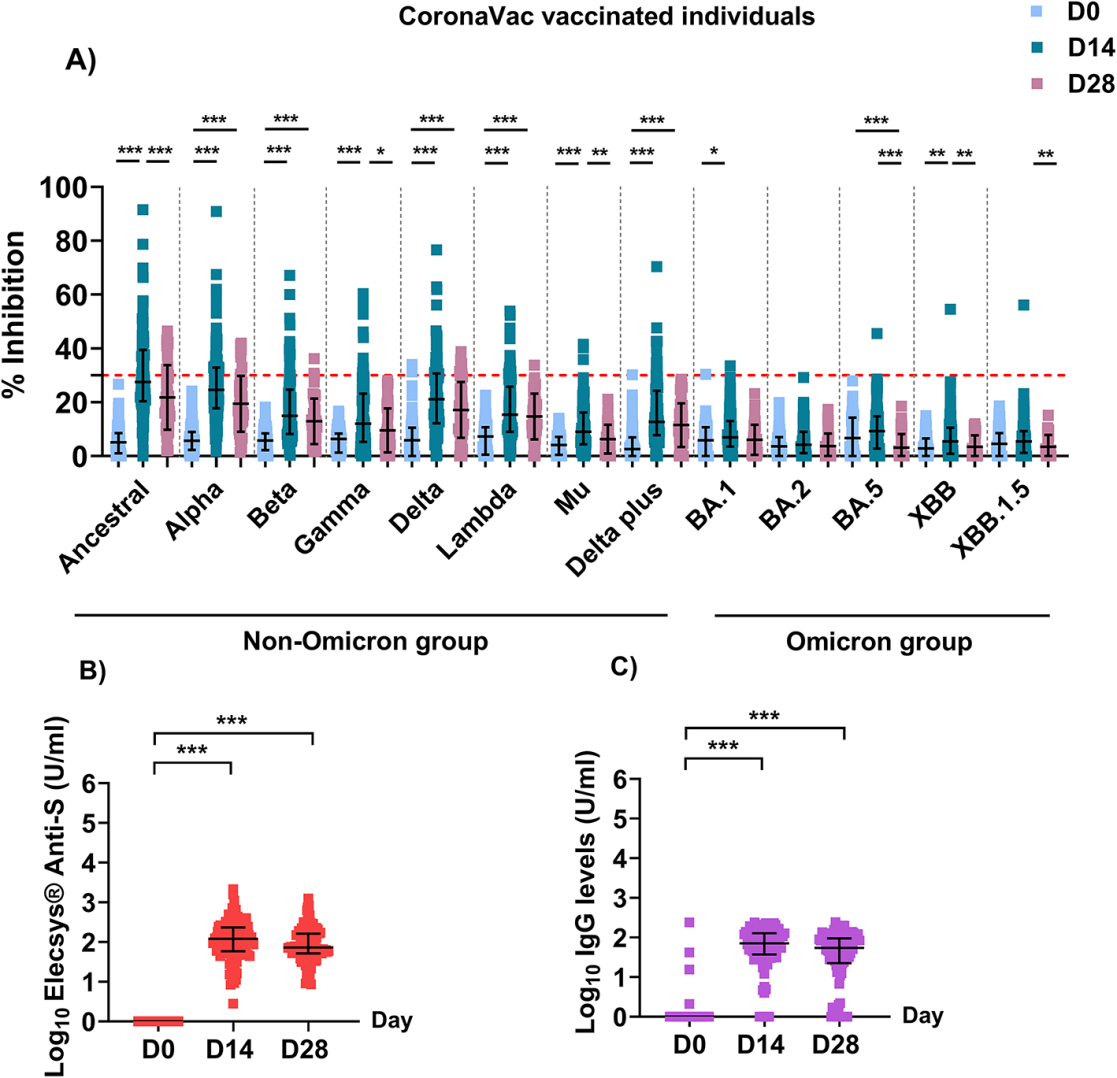
RBD-ACE2 blocking antibody (**A**), anti-spike antibody (**B**), and anti-RBD IgG antibody levels (**C**) in CoronaVac-vaccinated individuals. Blocking antibodies against ancestral SARS-CoV-2 and 12 non-Omicron and Omicron variants were determined using multiplex sVNT assay. Anti-spike antibodies were measured using the Elecsys^®^ anti-spike assay, and anti-RBD IgG antibodies against RBD of the Delta variant were determined using in-house ELISA. A negative result for nAbs was defined as a percent inhibition lower than 30% (indicated by the dotted red line), whereas a negative result for anti-spike antibodies was defined a level lower than 0.8 U/ml. The Kruksall-Wallis test with Dunn’s multiple comparison test was used to assess differences between groups. Data of anti-RBD IgG levels were obtained from^20^.

To characterize the antibody response after CoronaVac vaccination, which has a distinct antigenic composition compared to other vaccines and natural infection, we measured plasma RBD-ACE2 blocking antibody levels against various SARS-CoV-2 strains using multiplex sVNT^30,31^, anti-spike antibody levels using ECLIA^37^, and anti-RBD IgG antibody levels using in-house ELISA^20^ in vaccinated individuals on days 0 (pre-vaccination), 14, and 28 after the second dose (Fig. 2, Supplementary Tables S1–S3). All CoronaVac-vaccinated individuals displayed no evidence of prior SARS-CoV-2 infection at enrollment, as anti-nucleocapsid antibodies were negative on day 0 (cutoff index [COI] < 1.0) (Supplementary Fig. S1).

At baseline pre-vaccination, individuals had a median inhibition level of 5.7% against all SARS-CoV-2 strains on day 0, which was interpreted as negative (Fig. 2A). After CoronaVac vaccination, the median nAb levels for non-Omicron group significantly increased, reaching a median percent inhibition of 15.2% on day 14. However, nAb levels for Omicron group were only 5.5%. By day 28, nAbs levels had declined to 13.5% in the non-Omicron group and 1.4% in the Omicron group. The nAb activities against all SARS-CoV-2 strains remained below the cutoff for 28 days after CoronaVac vaccination.

In comparison, the median anti-spike antibody level as measured by ECLIA increased from 0.4 U/ml on day 0 to 121.6 U/ml on day 14 (*P* < 0.001), followed by a decline to 73.3 U/ml by day 28 (Fig. 2B).

Moreover, we measured the levels of anti-RBD IgG antibodies against the RBD of the Delta variant which was the dominant variant during our study, using ELISA. The median plasma antibody level significantly increased after two doses of CoronaVac from 0 U/ml on day 0 to 71.5 U/ml on day 14 (*P* < 0.001). Subsequently, the levels of anti-RBD IgG antibodies declined to 55.0 U/ml by day 28 (Fig. 2C).

### RBD-ACE2 blocking, anti-spike, and anti-RBD IgG antibody levels in COVID-19 patients

**Fig. 3:**
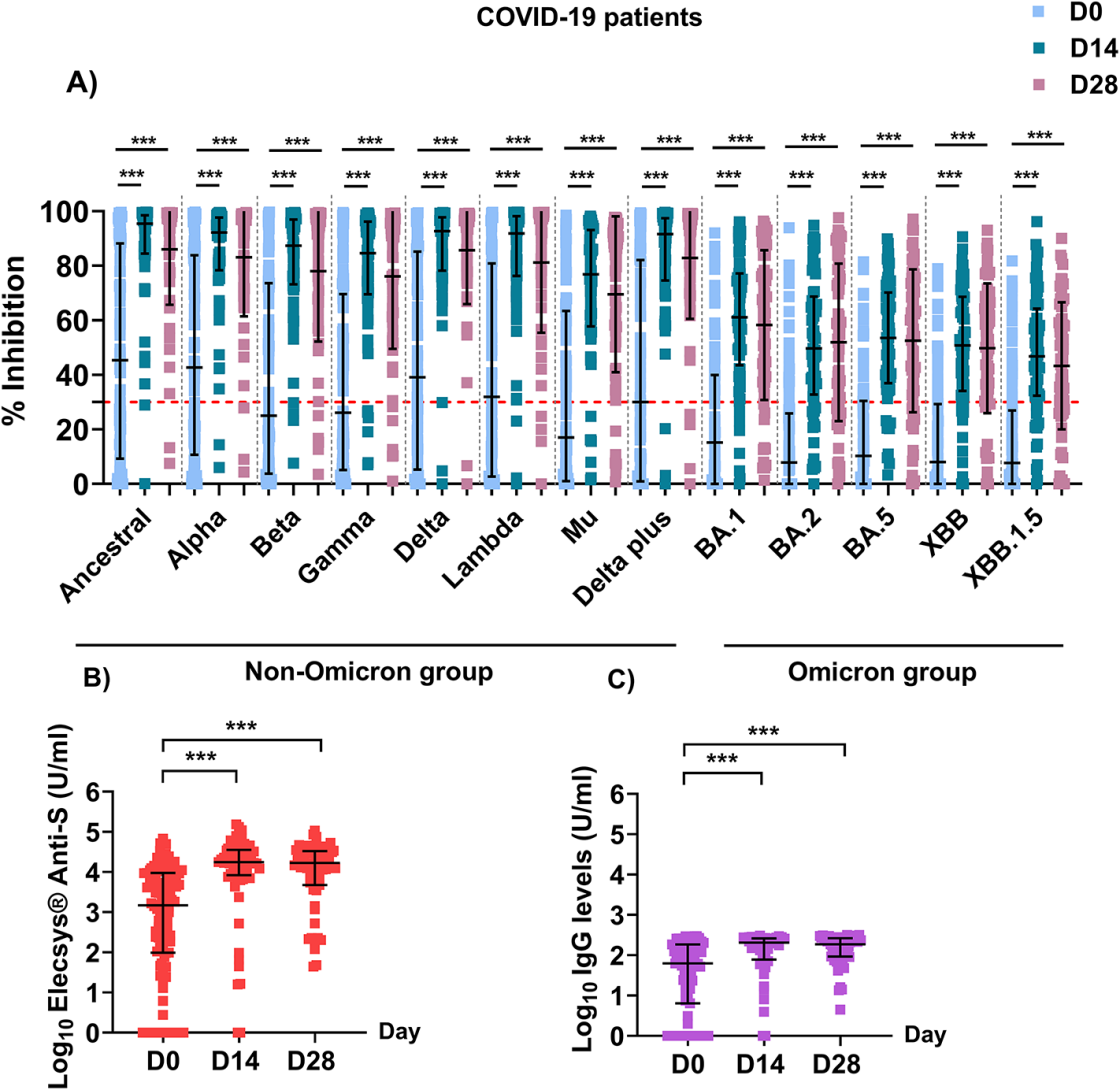
RBD-ACE2 blocking antibody (**A**), anti-spike antibody (**B**), and anti-RBD IgG antibody levels (C) in COVID-19 patients. Blocking antibodies against ancestral SARS-CoV-2 and 12 non-Omicron and Omicron variants were detected by multiplex sVNT, anti-spike antibodies were detected by the Elecsys^®^ anti-spike assay, and anti-RBD IgG antibodies against SARS-CoV-2 RBD of the Delta variant were detected by ELISA. A negative result for nAbs was defined as a percent inhibition lower than 30% (indicated by the dotted red line), whereas a negative result for anti-spike antibodies was defined levels lower than 0.8 U/ml. The Kruksall-Wallis test with Dunn’s multiple comparison test was used to assess differences between the groups. Data of nAb and anti-RBD IgG levels were obtained from^30,35^.

To understand the correlations among antibody levels in COVID-19 patients, we reanalyzed the initial blocking, anti-spike, and anti-RBD IgG levels in this study. The levels of blocking antibodies were higher in COVID-19 patients than in CoronaVac-vaccinated individuals on day 0 of study enrollment, with a median inhibition level of 22.7% against all SARS-CoV2 strain (Fig. 3A). Following infection, blocking antibody levels against all SARS-CoV-2 strains significantly increased by day 14, reach a median percent inhibition of 91.1% in the non-Omicron group (*P* < 0.001 for all strains) and 50.8% in the Omicron group (*P* < 0.001 for all strains). Blocking antibody levels persisted on day 28 compared with those on day 14, with a median percent inhibition of 93.6% in the non-Omicron group and 54.8% in the Omicron group.

Interestingly, anti-spike antibody levels were significantly increased on day 14, reaching a median of 17,777 U/ml (versus 1486 U/ml on day 0, *P* < 0.001). These levels remained high at 16,834 U/ml on day 28 (Fig. 3B).

The median anti-RBD IgG antibody level was significantly higher on day 14 (206.5 U/ml) than on day 0 (63 U/ml, *P* < 0.001). Its level remained elevated at 187.3 U/ml on day 28 (Fig. 3C).

Out of 111 COVID-19 patients, 94 breakthrough patients exhibited significantly higher levels of blocking antibodies against all SARS-CoV-2 strains at all time points compared to unvaccinated patients (*P* < 0.05 for all strains; Supplementary Fig. S2A–M). Similarly, anti-spike antibody levels were markedly elevated in breakthrough patients at all time points (*P* < 0.001; Supplementary Fig. S2N). However, anti-RBD IgG antibody levels were higher in breakthrough patients than in unvaccinated patients only on day 0 (*P* < 0.001; Supplementary Fig. S2O).We further compared antibody responses between unvaccinated COVID-19 patients and CoronaVac-vaccinated individuals (Supplementary Fig. S3). Interestingly, unvaccinated COVID-19 patients exhibited significantly higher levels of blocking antibodies than CoronaVac-vaccinated individuals on day 0 and day 28 (*P* < 0.05 for all strains; Supplementary Fig. S3A–M). However, anti-spike antibody levels were higher in unvaccinated patients than in CoronaVac-vaccinated individuals only on day 0 (*P* < 0.001; Supplementary Fig. S3N). Anti-RBD IgG antibody levels were higher in unvaccinated COVID-19 patients than in CoronaVac-vaccinated individuals on days 0 and 28 (*P* < 0.05; Supplementary Fig. S3O), suggesting that natural infection alone induces stronger immunity than two doses of CoronaVac vaccination.

### Correlation between RBD-ACE2 blocking and anti-spike antibody levels in CoronaVac-vaccinated individuals

**Fig. 4:**
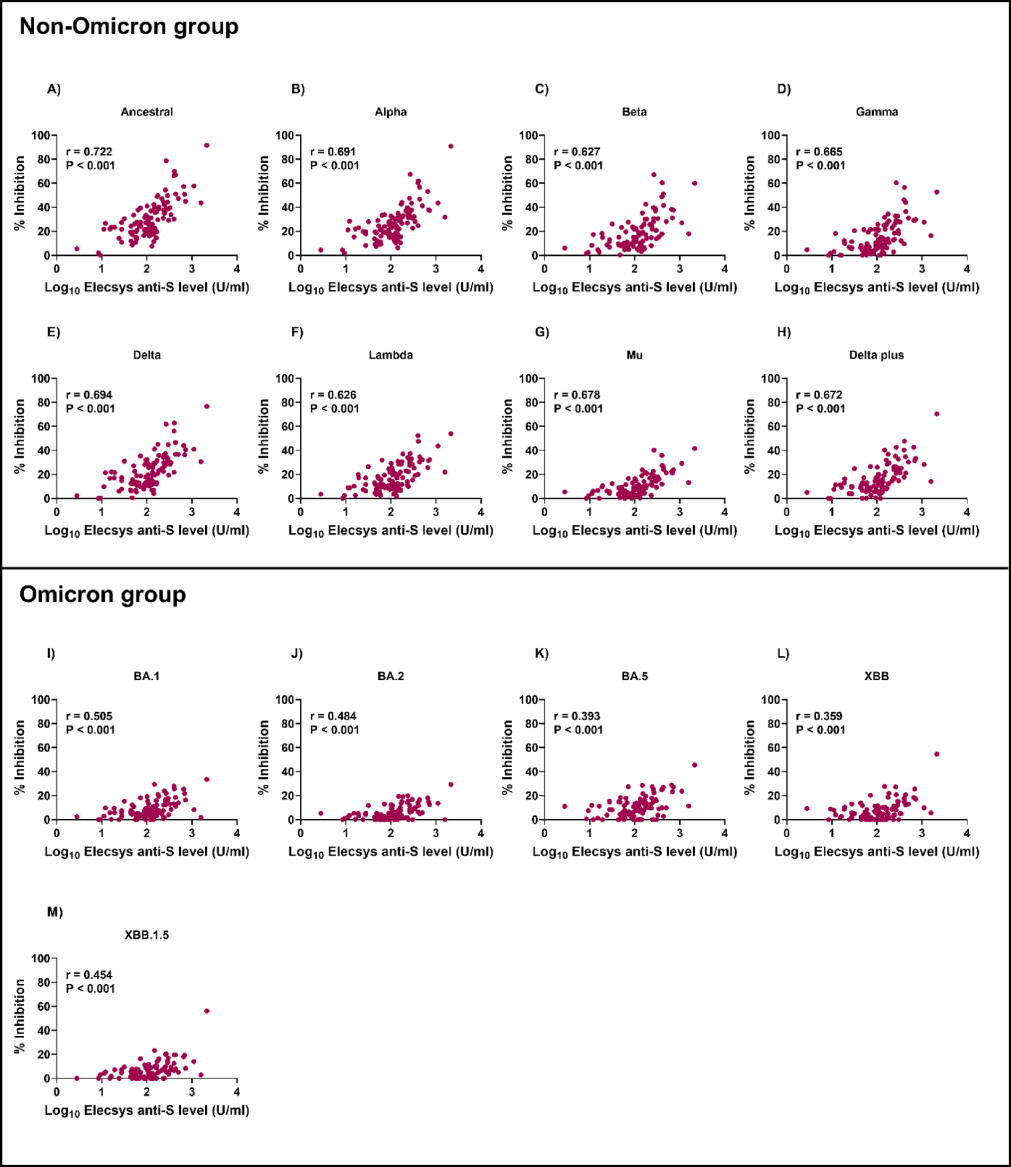
Correlation between RBD-ACE2 blocking and anti-spike antibody levels on day 14 in CoronaVac-vaccinated individuals. Blocking antibodies were detected using the sVNT assay, and anti-spike antibodies were detected using the Elecsys® anti-spike assay. The pairwise correlation coefficient (*r*) was determined using Spearman’s rank correlation.

**Fig. 5:**
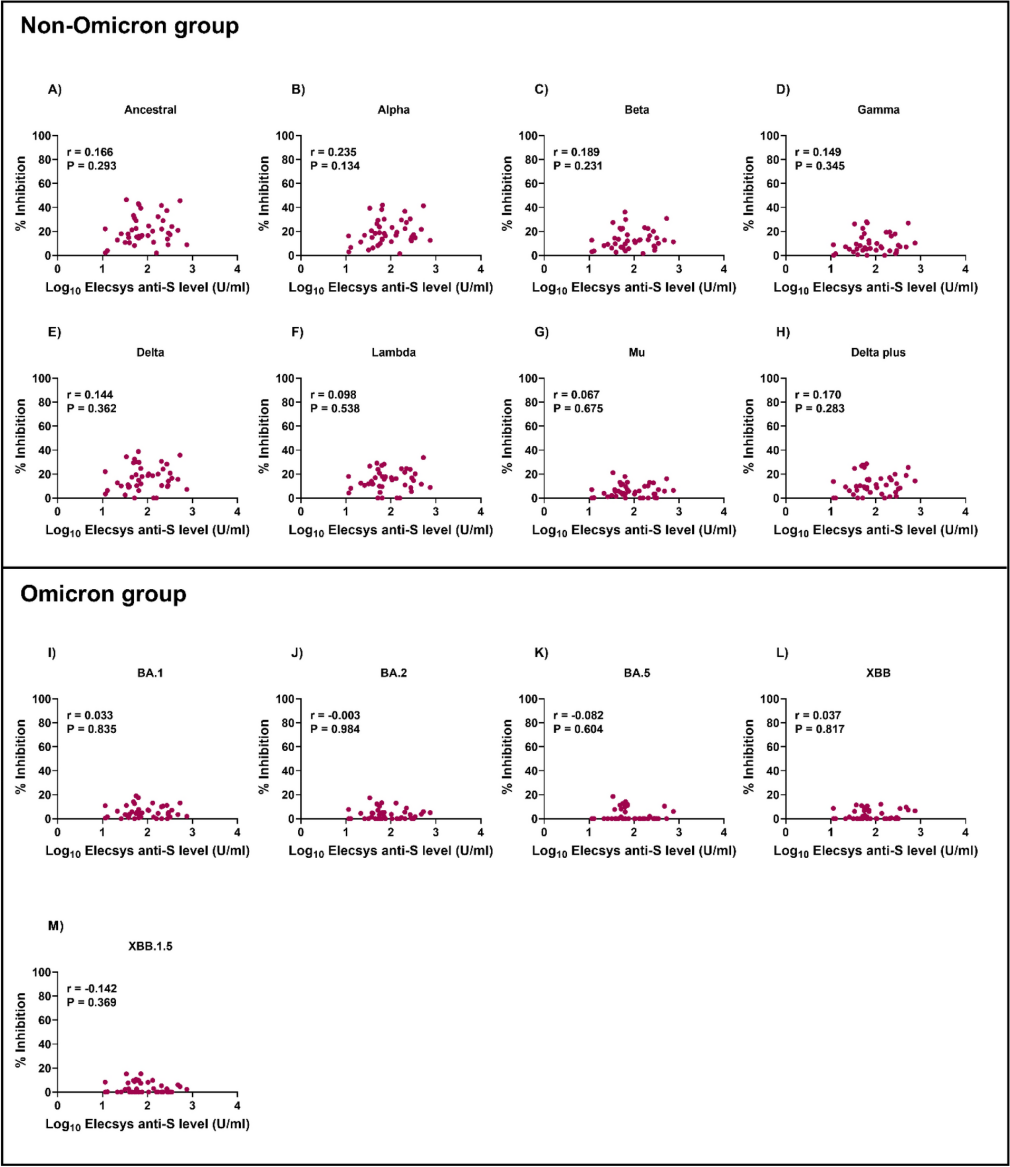
Correlations between RBD-ACE2 blocking and anti-spike antibody levels on day 28 in CoronaVac-vaccinated individuals. Blocking antibodies were detected using the sVNT assay, and anti-spike antibodies were detected using the Elecsys^®^ anti-spike assay. The pairwise correlation coefficient (*r*) was determined using Spearman’s rank correlation.

Blocking antibodies play a vital role in preventing viral entry into host cells, whereas anti-spike antibodies specifically target the spike protein on the virus surface and have a wider range of effector functions via wide interactions with the innate and adaptive immune systems. To understand the role of anti-spike antibodies induced by CoronaVac vaccination against different SARS-CoV-2 variants, we analyzed the correlation between blocking and anti-spike antibody levels in CoronaVac-vaccinated individuals (Figs. 4 and 5). We observed moderate correlations between blocking and anti-spike antibody levels on day 14 in the non-Omicron group (*r* = 0.626–0.722, *P* < 0.001 for all). However, these correlations were weak on day 28 (*r* = 0.067–0.235).

Conversely, on day 14, the correlation between blocking and anti-spike antibody levels was weaker for Omicron strains (*r* = 0.359–0.505, *P* < 0.001 for all). Interestingly, no correlation was observed for the Omicron group on day 28 (*r* =  − 0.142–0.037). These results suggest that the correlation between blocking antibody and anti-spike antibodies can vary over time and in response to different variants.

### Correlation between RBD-ACE2 blocking and anti-spike antibody levels in COVID-19 patients

**Fig. 6:**
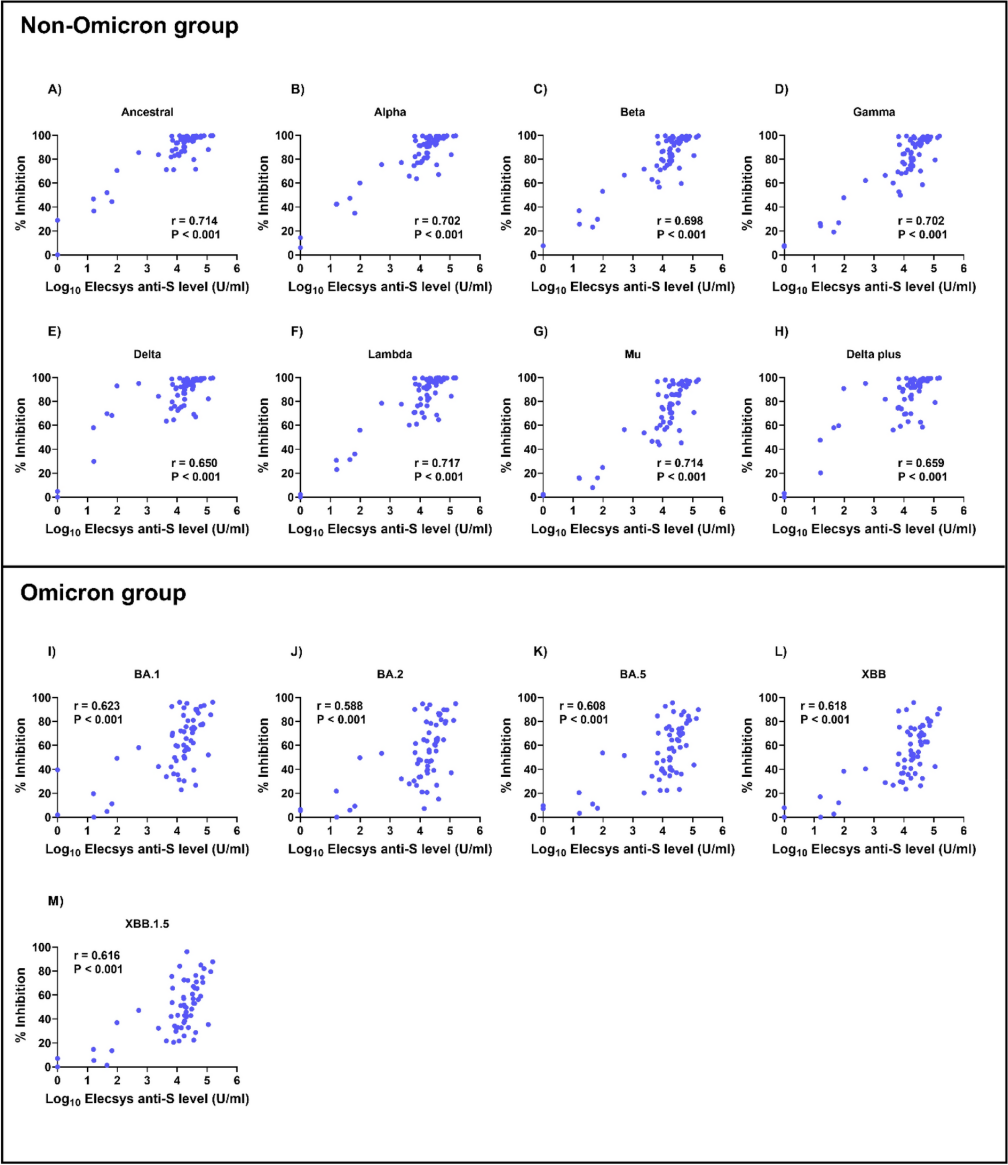
Correlation between RBD-ACE2 blocking and anti-spike antibody levels on day 14 in patients with COVID-19. Blocking antibodies were detected by the sVNT assay^30^, whereas anti-spike antibodies were measured using the Elecsys® anti-spike assay. The pairwise correlation coefficient (*r*) was determined using Spearman’s rank correlation.

**Fig. 7:**
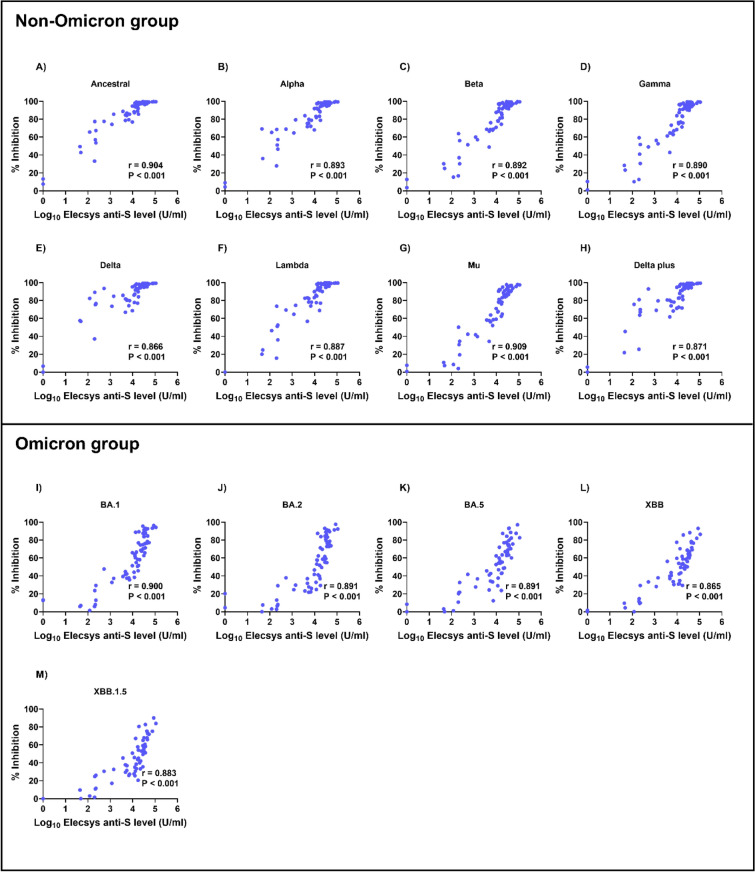
Correlation between RBD-ACE2 blocking and anti-spike antibody levels on day 28 in patients with COVID-19. Blocking antibodies were detected using the sVNT assay^30^, whereas anti-spike antibodies were measured using the Elecsys^®^ anti-spike assay. The pairwise correlation coefficient (*r*) was determined using Spearman’s rank correlation.

We analyzed the correlation between blocking antibody and anti-spike antibody levels in COVID-19 patients for different variants (Figs. 6 and 7). We observed a strong correlation between blocking antibody and anti-spike antibody levels on day 14 in the non-Omicron group (*r* = 0.650–0.717, *P* < 0.001 for all). Interestingly, the correlations between blocking and anti-spike antibody levels were significantly stronger on day 28 after enrollment (*r* = 0.866–0.909, *P* < 0.001 for all).

However, in the Omicron group, the correlation on day 14 was weaker (*r* = 0.588–0.623, *P* < 0.001 for all). Nevertheless, by day 28, the correlation was significantly stronger (*r* = 0.865–0.900, *P* < 0.001 for all).

### Correlation between anti-spike antibody and anti-RBD IgG antibody levels in CoronaVac-vaccinated individuals and COVID-19 patients

**Fig. 8:**
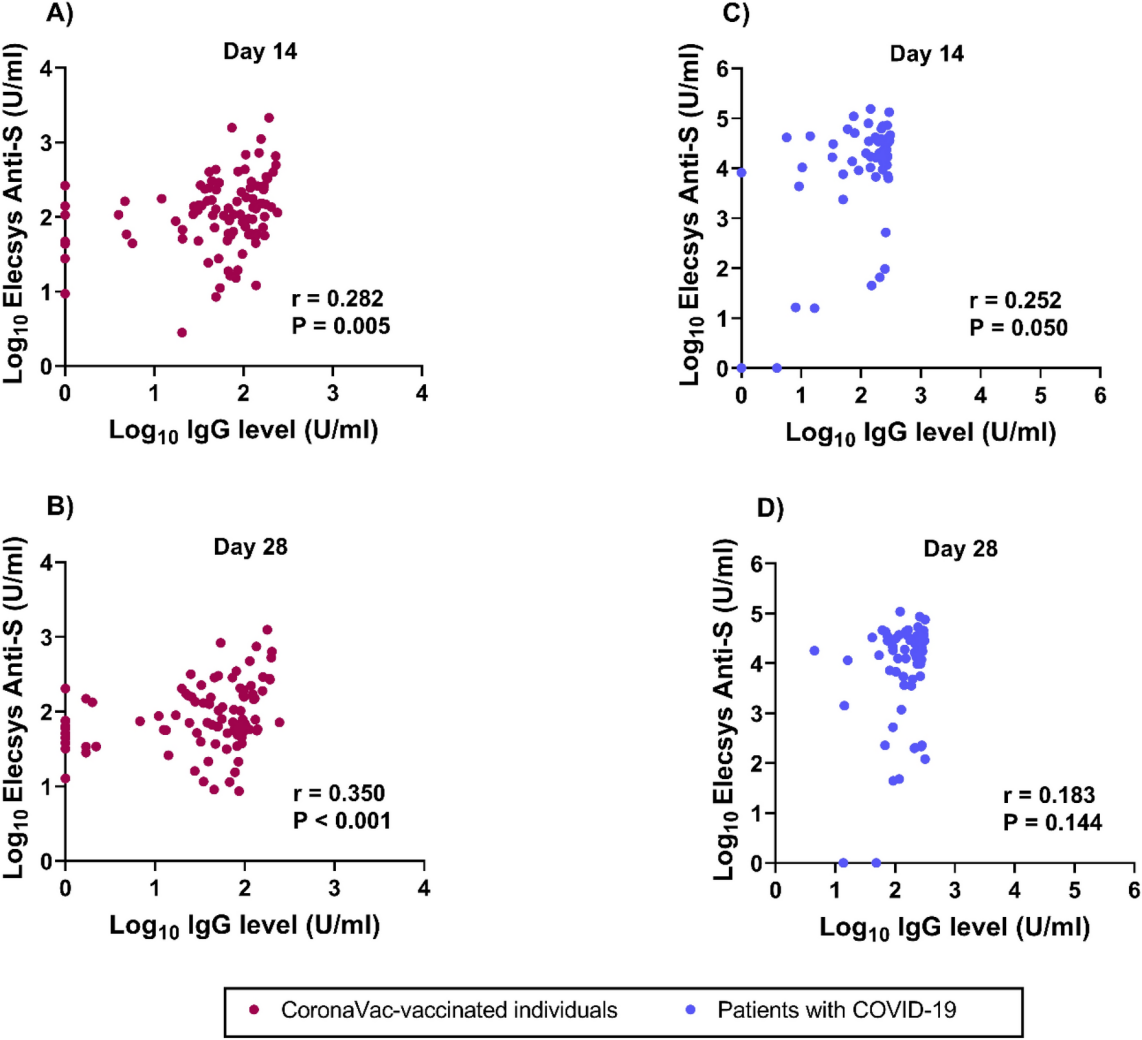
Correlation between anti-spike and anti-RBD IgG antibody levels on days 14 and 28 in CoronaVac-vaccinated individuals (A and B) and patients with COVID-19 (C and D). Anti-spike antibodies were detected using the Elecsys® anti-spike assay, and anti-RBD IgG antibodies against the SARS-CoV-2 Delta variant were measured using ELISA^20,35^. The pairwise correlation coefficient (*r*) was determined using Spearman’s rank correlation.

To evaluate the relationship between anti-spike and anti-RBD IgG antibodies, we performed ELISA as previously described^35^. Subsequently, we analyzed the correlation between anti-spike and anti-RBD IgG antibody levels. These two assays detected binding antibodies to the spike proteins (Fig. 8). We found a weak correlation between anti-spike and anti-RBD IgG antibody levels in CoronaVac-vaccinated individuals at all time points (day 14: *r* = 0.282, *P* = 0.005; day 28: *r* = 0.350, *P* < 0.001). The correlation was also low at all time points in COVID-19 patients (day 14: *r* = 0.252, *P* = 0.050; day 28: *r* = 0.183, *P* = 0.144).

### Correlation between RBD-ACE2 blocking and anti-RBD IgG antibody levels in CoronaVac-vaccinated individuals

We reanalyzed the anti-RBD IgG levels in CoronaVac-vaccinated individuals^20^ and found weak correlations between nAb and anti-RBD IgG antibody levels on day 14 in the non-Omicron group (*r* = 0.222–0.387, *P* < 0.050 for all, Supplementary Fig. S4). These correlations were slightly stronger on day 28 (*r* = 0.365–0.461, *P* < 0.050 for all, Supplementary Fig. S5).

The correlation in the Omicron group was weaker than that in the non-Omicron group on days 14 (*r* = 0.177–0.244) and 28 (*r* =  − 0.003–0.304). These findings suggest that the correlation between blocking and anti-RBD IgG antibody levels in vaccinated individuals decreases over time and varies in response to different SARS-CoV-2 variants.

### Correlation between RBD-ACE2 blocking and anti-RBD IgG antibody levels in COVID-19 patients

We analyzed the correlation between blocking and anti-RBD IgG antibody levels in COVID-19 patients. Unlike the correlation observed between blocking and anti-spike antibodies, weak correlations between their levels were observed in both the non-Omicron (*r* = 0.284–0.323, *P* < 0.050 for all) and Omicron groups (*r* = 0.266–0.294, *P* < 0.050 for all) on day 14 (Supplementary Fig. S6). The correlation remained low on day 28 in the non-Omicron (*r* = 0.036–0.203) and Omicron groups (*r* = 0.137–0.180, Supplementary Fig. S7). These results suggest that not all anti-RBD IgG antibodies had functional blocking activities.

## Discussion

This study investigated the correlations between RBD-ACE2 blocking antibodies, anti-spike antibodies, and anti-RBD IgG antibodies in CoronaVac-vaccinated individuals and COVID-19 patients in Thailand. All vaccinated individuals tested negative for anti-nucleocapsid antibody, confirming no prior SARS-CoV-2 infection. Among COVID-19 patients, 84.7% had breakthrough infections indicating a predominant “hybrid immunity” from a combination of ancestral-based vaccine followed by infection, primarily with Delta and Omicron subvariants, as most patients were recruited between July 2021 and December 2022.

CoronaVac vaccination induced measurable blocking antibody levels peaking on day 14, though with significant variability across variants and reduced activity against Omicron. Moderate correlation between RBD-ACE2 blocking and anti-spike antibody levels was observed on day 14, which weakened by day 28, while blocking and anti-RBD IgG antibody levels showed weak correlation at all time points. In contrast, COVID-19 patients exhibited significantly higher initial blocking antibody levels, likely due to greater antigen exposure from severe illness and hybrid immunity. Blocking levels varied across variants, with reduced effectiveness against Omicron. Anti-spike antibody levels were high on days 14 and 28, with a strong correlation between blocking and anti-spike antibody levels that increased over time. However, the correlation between blocking and anti-RBD IgG antibody levels remained low throughout.

Our finding aligns with previous studies in China and Cyprus reporting low antibody levels post-two doses of CoronaVac vaccination^38,39^. Antibody levels across all assays rapidly decreased by day 28, indicating a short-lived antibody response. CoronaVac is an inactivated whole SARS-CoV-2 virus vaccine with adjuvants, may induce different immune responses influenced by dosing and boosting schedules, population differences, and immunosenescence^40–43^. The difference in antibody levels in the two assays could be attributable to the differing principles of multiplex sVNT and ECLIA. Multiplex sVNT measures the inhibitory effects of blocking antibodies using multiplex beads coated with RBD proteins of the ancestral strain and its variants. Previous studies have shown that the inhibition of nAbs from sVNT were highly correlated with those from live virus neutralization^31–34^. However, ECLIA detects and quantifies total anti-spike antibodies by employing two spike proteins to form a double-antigen sandwich^24,37^. Our laboratory previously used indirect ELISA to detect anti-RBD IgG antibody levels. The present study clearly suggest that the anti-spike and anti-RBD IgG antibodies detected by ECLIA and ELISA may predict the presence of blocking antibodies, but not all anti-spike antibodies can neutralize SARS-CoV-2 variants^24^.

The reduced blocking activity against Omicron subvariants likely results from extensive spike mutations, enhancing immune escape^44,45^. Immune imprinting may also contribute, as CoronaVac vaccination induces responses based on ancestral strains, potentially limiting nAb effectiveness against emerging variants^46^. Previous studies have demonstrated that three doses of CoronaVac elicit broader antibody and T cell responses^47,48^. T cell immunity plays a crucial role in recognizing conserved viral regions, providing durable cross reactivity against highly mutated subvariants like BA.2.86 compared to humoral immunity^49^.

Booster doses represent a strategy to enhance and prolong immunity, as two doses of CoronaVac at 3 weeks apart may not generate a robust or long-lasting response against new emerging variants. The World Health Organization (WHO) now recommends incorporating the JN.1 lineage into vaccine formulations to enhance protection (https://www.who.int/). Omicron-specific and bivalent vaccines have demonstrated superior neutralization activity against Omicron subvariants in both animal models and vaccinated individuals compared to ancestral-strain vaccines^50,51^.

COVID-19 patients exhibited strong blocking antibody activity, with nearly 100% inhibition for the non-Omicron group, but approximately 50% for Omicron group^30^. The levels of anti-spike and anti-RBD IgG antibodies were significantly increased and remained elevated^35^. The complex immune response induced by natural infection likely results from prolonged antigen exposure, driving robust activation of CD4 and CD8 T cells, natural killer (NK) cells, memory B cells, and nAbs^52^. Our results were consistent with previous studies reporting higher nAb levels against non-Omicron variants and lower levels against Omicron^53^. The slow decline and persistence of anti-spike and anti-RBD IgG antibodies for several months after infection compared with vaccination are consistent with previous observations^54,55^.

Vaccine-induced antibodies mainly target the spike protein but may wane over time, whereas natural infection generates broader antibodies with potential cross-protection. Additionally, natural infection provides longer antigen exposure than vaccination, lasting days to weeks and leading to sustained immune activation. Moreover, multiple infections can induce broader and stronger neutralizing activity compared to a primary infection^49,52^. However, their durability and neutralization capacity vary among individuals^47–49^. Studying unexposed individuals has become challenging, but recent research highlights Syrian hamsters as a surrogate model for generating variant-specific sera, revealing substantial antigenic diversity among Omicron subvariants^56^. This aligns with our findings of varying antibody responses to different variants in CoronaVac-vaccinated individuals and COVID-19 patients.

After CoronaVac vaccination, the moderate correlations between blocking and anti-spike antibody levels on day 14, and the lack of significant correlation on day 28, suggest a waning of the antibody-based immune response over time. No prior studies have reported RBD-ACE2 blocking and anti-spike antibody correlations in CoronaVac recipients though stronger correlations have been observed in mRNA recipients and heterologous vaccine regimens^26,27^.

The limited induction of nAbs by CoronaVac may reduce its effectiveness in viral clearance, impacting overall control of virus spread^40–42,57^. Our study suggests that the timing of antibody assessment post-vaccination could affect the correlation between blocking and anti-spike antibody levels, as antibody levels can decline at different rates among individuals^58^. Moreover, not all anti-spike antibodies confer blocking SARS-CoV-2 variants^24,31,37^.

At enrollment, patients had a median of 1 day (0–2) since hospital admission. A strong correlation between blocking and anti-spike antibody levels was observed, strengthened from day 14 to day 28, indicating a sustained antibody-based immune response post-infection, consistent with previous studies^24,59^. During the early stage of infection, the antibody-based immune response may not be fully mature^44,45,52^. Moreover, treatments such as corticosteroids, used to reduce mortality in severe COVID-19 cases, may modulate the antibody response^60,61^.

The stronger correlation between blocking and anti-spike antibody levels on day 28 compared to day 14 aligns with prior findings of a more robust response in COVID-19 patients versus vaccinated individuals^20,25^. This suggests that the antibody response takes several days post-infection to mature, allowing a more effective and specific response against variants^52^. The maturation of immune cells significantly contributes to immune function maintenance^62^, with blocking antibody levels typically peaking around 2 weeks post-symptom onset, persisting up to 4 weeks before stabilizing^63^.

Low correlation between blocking and anti-RBD IgG antibody levels against all SARS-CoV-2 strains at all time points in both CoronaVac-vaccinated individuals and COVID-19 patients contrasts previous research reporting stronger correlations^29^. The difference in correlations potentially due to variations in different ELISAs sensitivity^32,37,64^. Additionally, IgG antibodies target a broad range of epitopes, potentially binding to non-neutralizing epitopes^65^. Moreover, IgG subclasses (IgG1, IgG2, IgG3, and IgG4) also vary in neutralize capacity^36,66^. ELISA measures total IgG without distinguishing between these subclasses, potentially explaining the presence of non-neutralizing antibodies^20^.

Limitations of this study included the participant dropout, home isolation policies, relocation, and travel challenges. Additionally, the study did not include individuals who had received three doses of CoronaVac vaccine, as well as the bivalent vaccine. Timelines for vaccinated individuals and COVID-19 patients are not directly comparable. Vaccinated individuals were exposed only to the ancestral-variant-based CoronaVac vaccine, while most COVID-19 patients had Omicron infections post-vaccination. In addition, multiplex sVNT measured RBD-ACE2 blocking activity only, and the B cell response was not assessed in this study. Future studies should explore T cell responses and different vaccine regimens to further elucidate immune responses to SARS-CoV-2 variants. Additionally, memory B-cell responses should be further studied to assess long-term immunity. However, our findings underscore the need for ongoing serological surveillance, booster doses, and potentially updated vaccines to maintain protection against evolving SARS-CoV-2 variants. This information is essential for future responses to new pandemics.

## Materials and methods

### Ethics statement

This study was approved by the Ethics Committees of the Faculty of Tropical Medicine, Mahidol University (MUTM 2021-028-01 and MUTM 2021-019-01) and the Faculty of Medicine, Ramathibodi Hospital (MURA 2021/264). The research adhered to the ethical principles outlined in the Declaration of Helsinki (2008) and the International Conference on Harmonization Good Clinical Practice guidelines. Written informed consent was obtained from all participants prior to enrollment.

### Enrollment of vaccinated individuals

One hundred eleven individuals were examined on days 0 (pre-vaccination), 14, and 28 after receiving two doses of CoronaVac at the Hospital for Tropical Diseases (Bangkok, Thailand) between May and June of 2021 as previously described^20^. The interval between the first and second doses was 21–28 days. The eligibility criteria for vaccinated participants included healthy Thai adults aged 18 years or older who were capable of providing informed consent. The exclusion criteria were pregnancy or delivery within the preceding 9 months; the use of immune-modifying agents, anti-inflammatory agents, or cell-depleting biological agents in the last 4 weeks; the presence of a chronic medical condition, vigorous exercise in the past 24 h; alcohol consumption in the past 24 h; the receipt of immunoglobulins or blood products in the past 4 months; vaccination in the last month; the receipt of any research medicines or research vaccines in the past month; planned surgery within 6 months after the last immunization; or a history of COVID-19 infection. Baseline control samples were collected before the administration of the initial vaccination dose on day 0 for all immunoassays. Details of the vaccination history and the timeline of sample collections are shown in Table 1 and Fig. 1A.

### Enrollment of COVID-19 patients

One hundred eleven patients aged ≥ 18 years old with RT-PCR positive for the open reading frame 1ab gene (which encodes non-structural proteins) and the nucleocapsid gene of COVID-19 were enrolled at the Hospital for Tropical Diseases, Faculty of Tropical Medicine, Mahidol University between July 2021 and December 2022 (n = 88), Vichaivej Hospital, Samutsakhon Province between February 2022 and March 2022 (n = 7), and Prachathipat Hospital, Pathum Thani Province between March 2022 and August 2022 (n = 16) as previously described^30^. The exclusion criteria were pregnancy or delivery in the past 9 months; and the use of immuno-modifying, anti-inflammatory, and cell-depleting biological agents in the past 4 weeks. Patients were enrolled into our study a median of 1 day (range 0–2) post hospital admission with COVID-19, and plasma samples were collected at enrollment (day 0, n = 111), day14 (n = 65) and day 28 (n = 65) days later. Plasma samples were stored at − 80 °C. During enrollment, 94 out of 111 patients had received vaccines prior to infection, as shown in Table 1 and Fig. 1A.

### The antigenic composition of CoronaVac

CoronaVac is composed of inactivated whole SARS-CoV-2 virus, with aluminum hydroxide, disodium hydrogen phosphate, sodium dihydrogen phosphate, sodium chloride, and water for injection (https://www.who.int/publications/i/item/WHO-2019-nCoV-vaccines-SAGE_recommendation-Sinovac-CoronaVac-background-2021.1).

### sVNT

RBD-ACE2 blocking antibodies in the plasma of CoronaVac-vaccinated individuals and COVID-19 patients were measured using sVNT^30^. sVNT uses a non-infectious pseudovirus system to measure the ability of antibodies to block the interaction between the SARS-CoV-2 spike protein and ACE2, thus mimicking the virus neutralization process^64^. Plasma samples were diluted in 1% bovine serum albumin/PBS/1 M NaCl buffer, mixed with 13-plex RBD-conjugated MagPlex beads (1:200 final dilution), and incubated in a 96-well plate at 25 °C for 1 h. After incubation, 50 µl of huACE2-PE conjugate (1000 ng/ml) were added and incubated for 1 h. After discarding the contents of the plate, it was washed, and the mixture was then resuspended in 75 µl of assay buffer before performing the measurement. cPass SARS-CoV-2 positive control (GenScript, Piscataway, NJ, USA) and assay buffer served as positive and negative controls, respectively. The fluorescence intensity was determined using a MAGPIX® and presented as the inhibition percentage, which was calculated as median fluorescent intensity of test samples × 100/median fluorescent intensity of negative samples. The percentage inhibition was interpreted as indicative of RBD neutralizing activity.

### ECLIA

Elecsys^®^ Anti-SARS-CoV-2 S (anti-RBD) and Elecsys^®^ Anti-SARS-CoV-2 (anti-N) assays (Roche, Basel, Switzerland) were employed to detect total antibodies specific to the recombinant spike and nucleocapsid proteins of SARS-CoV-2. ECLIA is based on the principle of electrochemiluminescence, in which antibodies bound to ancestral SARS-CoV-2 spike antigens on an electrode surface emit light upon excitation by an electrical current (Roche Diagnostics International Ltd, Rotkreuz, Switzerland). The assay kit utilized recombinant proteins capable of detecting infections caused by all SARS-CoV-2 variants, as specified by the manufacturer. To determine anti-SARS-CoV-2 spike and nucleocapsid levels, plasma samples was analyzed using a double-antigen sandwich assay format, allowing incubation with biotinylated SARS-CoV-2–specific recombinant spike or nucleocapsid protein and the same proteins tagged with a ruthenium complex to form a sandwich complex with plasma samples. Streptavidin-coated microparticles were then added to facilitate binding of the complex to the solid phase via a biotin–streptavidin interaction. The reaction mixture was transferred to the measuring cell, in which an electrode applied voltage, leading to the generation of chemiluminescent emission. The emitted light was subsequently measured and analyzed using a Cobas e411 analyzer. The determination of anti-spike antibodies was performed in both vaccinated individuals and patients with COVID-19, whereas anti-nucleocapsid antibodies were detected to confirm no prior SARS-CoV-2 infection before vaccination in vaccinated individuals on day 0.

A 1:100 dilution was used to detect Elecsys^®^ Anti-SARS-CoV-2 S in all samples. For samples with values exceeding 25,000 U/ml, a 1:300 dilution was applied, and for values exceeding 75,000 U/ml, a 1:600 dilution was used. The results were interpreted by comparing the electrochemiluminescence signal from plasma samples with that from the calibration’s cutoff. Anti-SARS-CoV-2 S (anti-RBD) antibody levels were calculated as U/ml, whereas anti-SARS-CoV-2 (anti-N) antibody levels were calculated using COI. Non-reactive results were indicated by < 0.8 U/ml for anti-spike antibody and COI < 1.0 for anti-nucleocapsid antibody. To ensure experiment accuracy and reliability, we used an internal control supplied by the manufacturer and positive controls generated by pooling plasma samples from 10 vaccinated individuals. Each sample was analyzed once in duplicate.

### ELISA

The levels of IgG antibodies specific to the recombinant RBD of the SARS-CoV-2 Delta variants [Arg319-Ser591([L452R, T478K)] were determined in plasma samples from individuals vaccinated with CoronaVac and COVID-19 patients using in-house ELISA, as previously described^20,35,36^. Plasma samples were diluted to 1:100, and secondary antibodies and horseradish peroxidase-conjugated anti-human IgG (Dako, Glostrup, Denmark) were used at a dilution of 1:4000. Positive and negative controls were incorporated into each assay plate, including pooled plasma samples from 10 patients with RT-PCR-confirmed COVID-19 and 10 pre-vaccinated healthy donors. Additionally, the assay diluent was consistently applied, and the values were calculated as the mean ± standard deviation. The unit calculation was based on an optical density (OD) value of 0.01, which was interpreted as antibody level of 1 U/ml.

### Statistical analysis

All data were analyzed using GraphPad Prism version 8.0 (GraphPad Software Inc., San Diego, CA, USA). The Kruksall-Wallis test with Dunn’s multiple comparison test was used to assess differences between groups, and Spearman’s correlation was utilized to test the correlations between groups. P < 0.05 was considered statistically significant.

## Supplementary Information

Supplementary Information.

## Data Availability

All data generated or analyzed in this study are included in this published article and its supplementary information files.
