# Supplementary material for: Temporal correlations between RBD-ACE2 blocking and binding antibodies to SARS-CoV-2 variants in CoronaVac-vaccinated individuals and their persistence in COVID-19 patients
Source: Sci Rep. 2025 May 6;15:15831. doi: 10.1038/s41598-025-98627-3 (PMC12056071; doi:10.1038/s41598-025-98627-3)
Supplement: Supplementary file 1 — Supplementary Information. [file 41598_2025_98627_MOESM1_ESM.docx]

**Temporal correlations between** **RBD-ACE2 blocking and binding antibodies to SARS-CoV-2 variants in CoronaVac-vaccinated individuals and their persistence in COVID-19 patients**

Prapassorn Poolchanuan^a^, Wasin Matsee^b,c^, Adul Dulsuk^a^, Rungnapa Phunpang^a^, Chakkaphan Runcharoen^d,e^, Thitiya Boonprakob^f^, Onura Hemtong^f^, Suchada Chowplijit^g^, Vachara Chuapaknam^g^, Tanaya Siripoon^b,n^, Phimphan Pisutsan^b,c^, Watcharapong Piyaphanee^b,c^, Wathusiri Khongsiri^a^, Nathamon Kosoltanapiwat^a^, Le Van Tan^h,i^, Susanna Dunachie^j,k^, Chee Wah Tan^l,m^, Lin-Fa Wang^l^, Wasun Chantratita^d^, Viravarn Luvira^b,n^, Narisara Chantratita^a,k^, On behalf of the SEACOVARIANTS

^a^Department of Microbiology and Immunology, Faculty of Tropical Medicine, Mahidol University, Thailand

^b^Department of Clinical Tropical Medicine, Faculty of Tropical Medicine, Mahidol University, Thailand

^c^Thai Travel Clinic, Hospital for Tropical Diseases, Faculty of Tropical Medicine, Mahidol University, Thailand

^d^Center for Medical Genomics, Faculty of Medicine Ramathibodi Hospital, Mahidol University, Thailand

^e^Faculty of Medical Technology, Huachiew Chalermprakiet University, Thailand

^f^Prachatipat Hospital, Pathum Thani, Thailand

^g^Vichaivej International Hospital, Samut Sakhon, Thailand

^h^Oxford University Clinical Research Unit, Ho Chi Minh City, Vietnam

^i^Centre for Tropical Medicine and Global Health University of Oxford, United Kingdom

^j^NDM Centre for Global Health Research, Nuffield Department of Clinical Medicine, University of Oxford, Oxford, United Kingdom

^k^Mahidol-Oxford Tropical Medicine Research Unit, Faculty of Tropical Medicine, Mahidol University, Thailand

^l^Programme in Emerging Infectious Diseases, Duke-NUS Medical School, Singapore

^m^Infectious Diseases Translational Research Programme, Department of Microbiology and Immunology, Yong Loo Lin School of Medicine, National University of Singapore, Singapore

^n^Vaccine Trial Centre, Faculty of Tropical Medicine, Mahidol University, Thailand

**Corresponding autho**r: Narisara Chantratita, Department of Microbiology and Immunology, Faculty of Tropical Medicine, Mahidol University, 420/6 Rajvithi Road, Bangkok 10400, Thailand. E-mail: [narisara@tropmedres.ac](mailto:narisara@tropmedres.ac)

**Supplementary data**


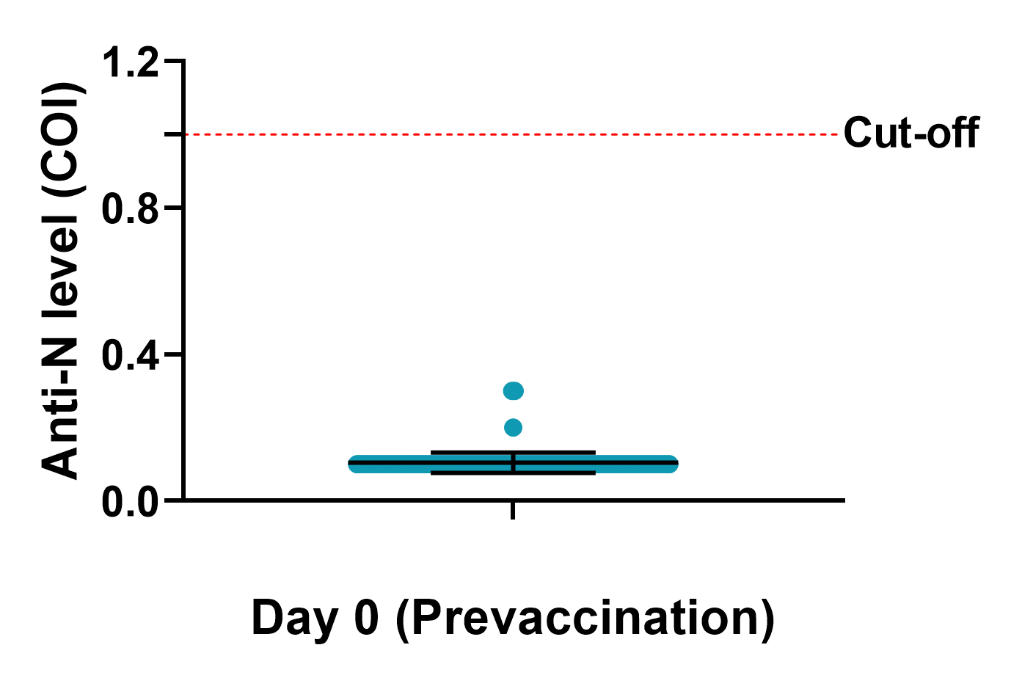


Supplementary Figure S1 Anti-nucleocapsid (N) antibody to SARS-CoV-2 in the plasma of 111 pre-vaccinated individuals on day 0. The anti-N antibody was determined by ECLIA assay. The non-reactive results were a value range of cut-off index (COI) < 1.0.


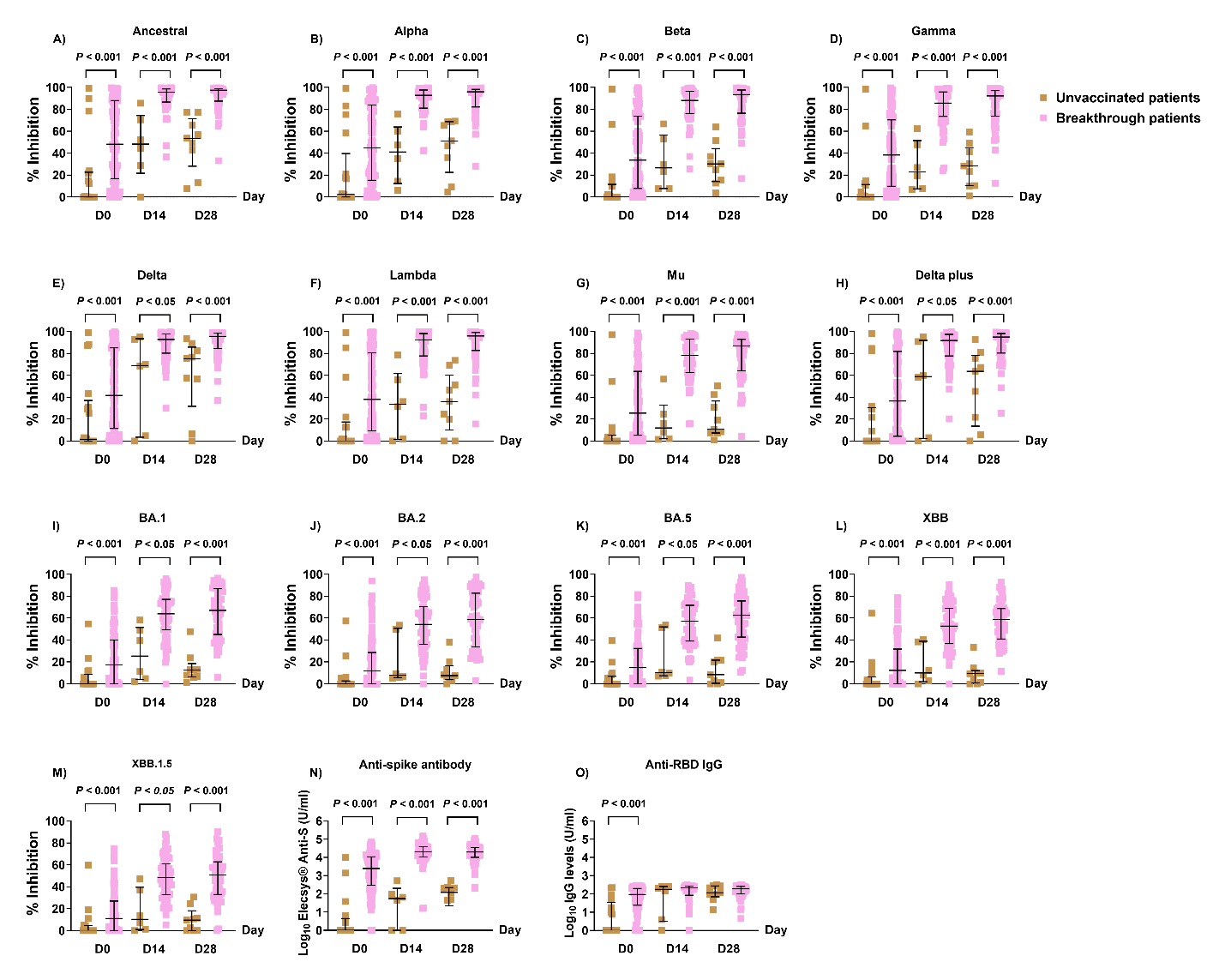


**Supplementary Figure S2** RBD-ACE2 blocking antibody (A-M), anti-spike antibody (N), and anti-RBD IgG antibody levels (O) in unvaccinated (N = 17) and breakthrough COVID-19 patients (N = 94). Blocking antibodies against ancestral SARS-CoV-2 and 12 non-Omicron and Omicron variants were detected by multiplex sVNT, anti-spike antibodies were detected by the Elecsys® anti-spike assay, and anti-RBD IgG antibodies against SARS-CoV-2 RBD of the Delta variant were detected by ELISA. A negative result for nAbs was defined as a percent inhibition lower than 30%, whereas a negative result for anti-spike antibodies was defined levels lower than 0.8 U/ml. The Mann-Whitney test was used to assess differences between the groups. Data of nAb and anti-RBD IgG levels were obtained from (30, 35).


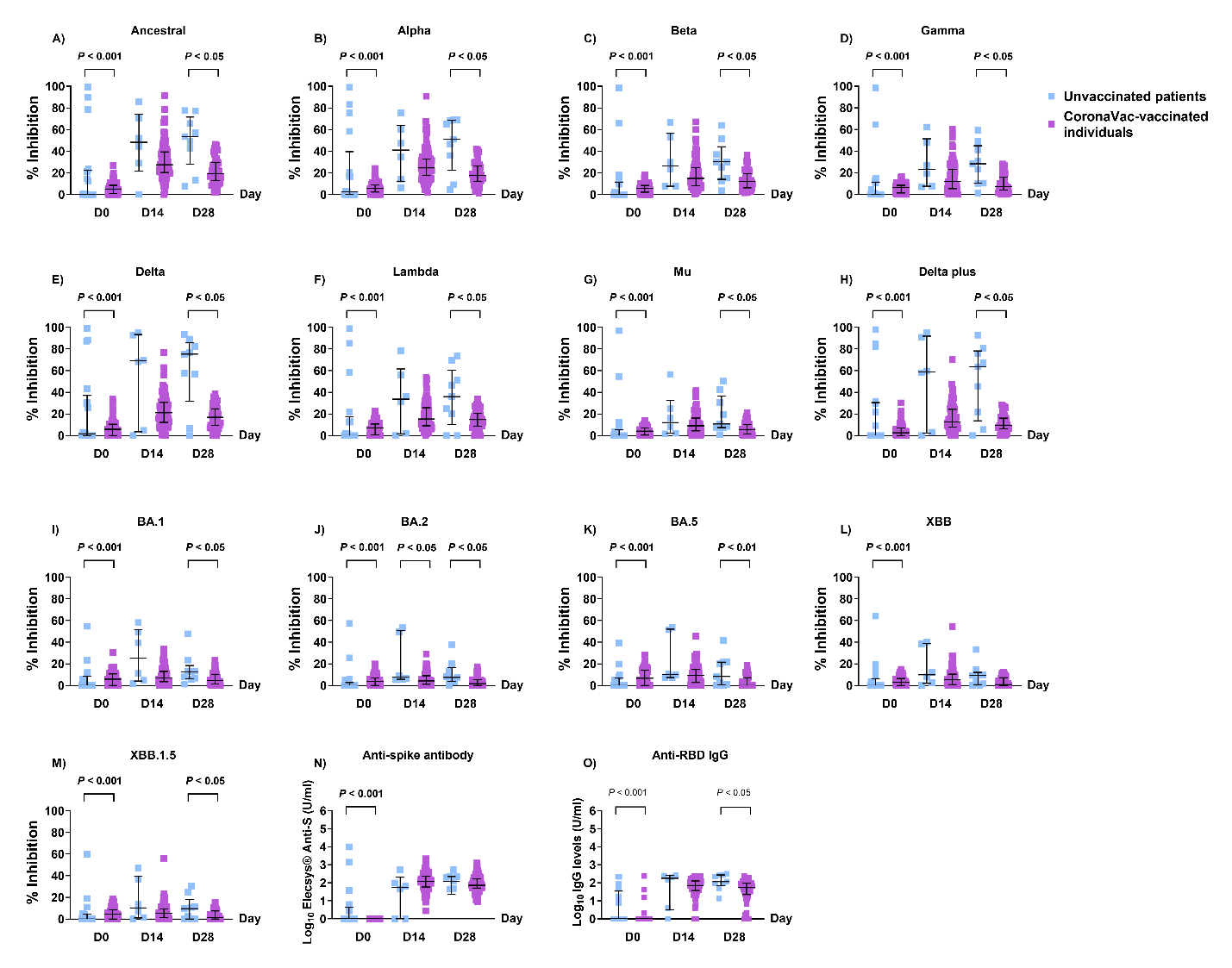


**Supplementary Figure S3** RBD-ACE2 blocking antibody (A-M), anti-spike antibody (N), and anti-RBD IgG antibody levels (O) in unvaccinated COVID-19 patients (N = 17) and CoronaVac-vaccinated individuals (N = 111). Blocking antibodies against ancestral SARS-CoV-2 and 12 non-Omicron and Omicron variants were detected by multiplex sVNT, anti-spike antibodies were detected by the Elecsys® anti-spike assay, and anti-RBD IgG antibodies against SARS-CoV-2 RBD of the Delta variant were detected by ELISA. A negative result for nAbs was defined as a percent inhibition lower than 30%, whereas a negative result for anti-spike antibodies was defined levels lower than 0.8 U/ml. The Mann-Whitney test was used to assess differences between the groups. Data of nAb and anti-RBD IgG levels were obtained from (30, 35).


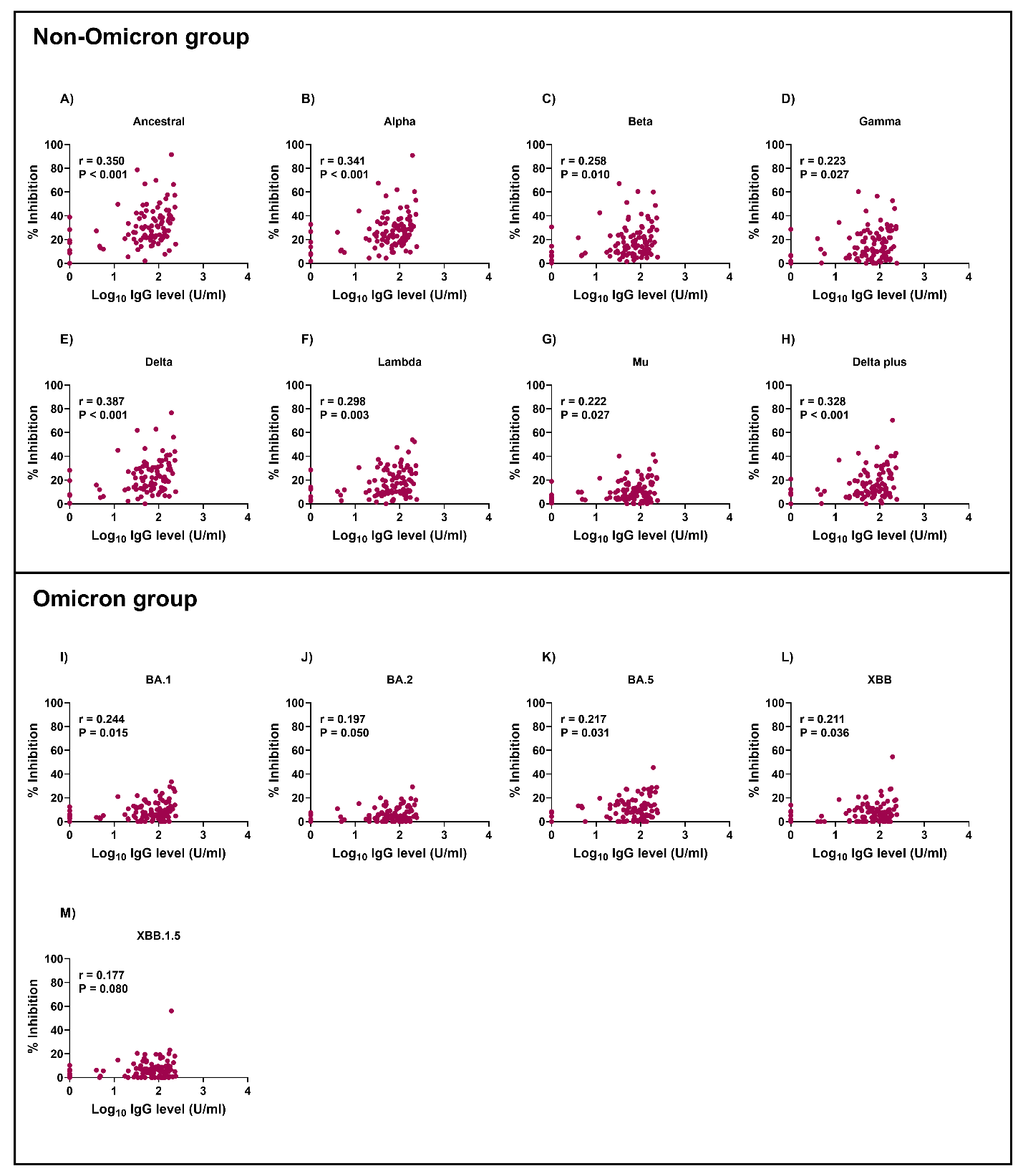


**Supplementary Figure S4** Correlation between RBD-ACE2 blocking and anti-spike IgG antibody levels on day 14 in CoronaVac-vaccinated individuals. Blocking antibodies were determined using the sVNT assays (30) (Poolchanuan et al., submitted for publication), and IgG antibodies against the RBD of the SARS-CoV-2 delta variant were determined using ELISA (35). The pairwise correlation coefficient (*r*) was determined using Spearman’s rank correlation.

**
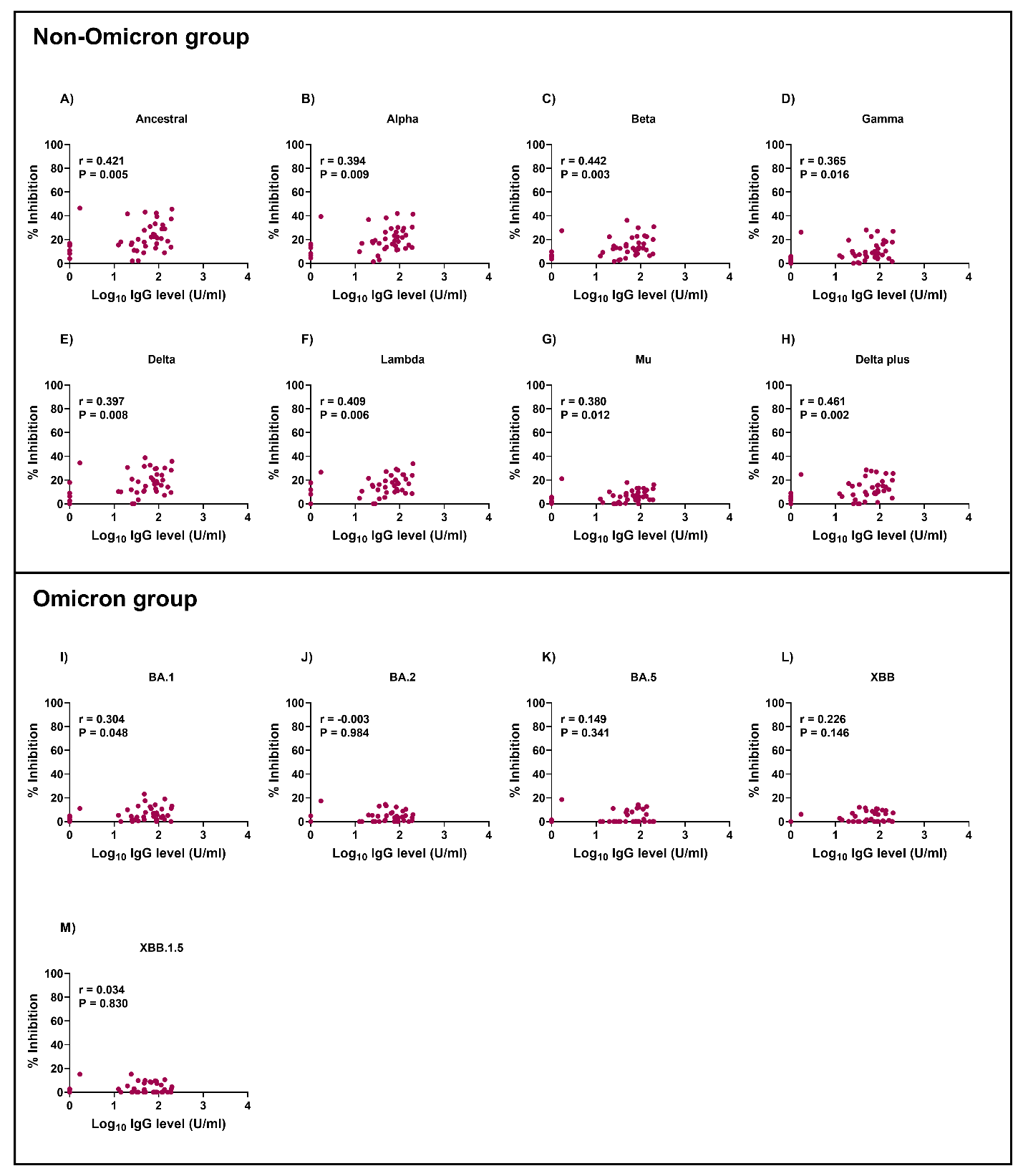
**

**Supplementary Figure S5** Correlation between RBD-ACE2 blocking and anti-spike IgG antibody levels on day 28 in CoronaVac-vaccinated individuals. Blocking antibodies were determined using the sVNT assays (30) (Poolchanuan et al., submitted for publication), and IgG antibodies against the RBD of the SARS-CoV-2 delta variant were determined using ELISA (35). The pairwise correlation coefficient (*r*) was determined using Spearman’s rank correlation.


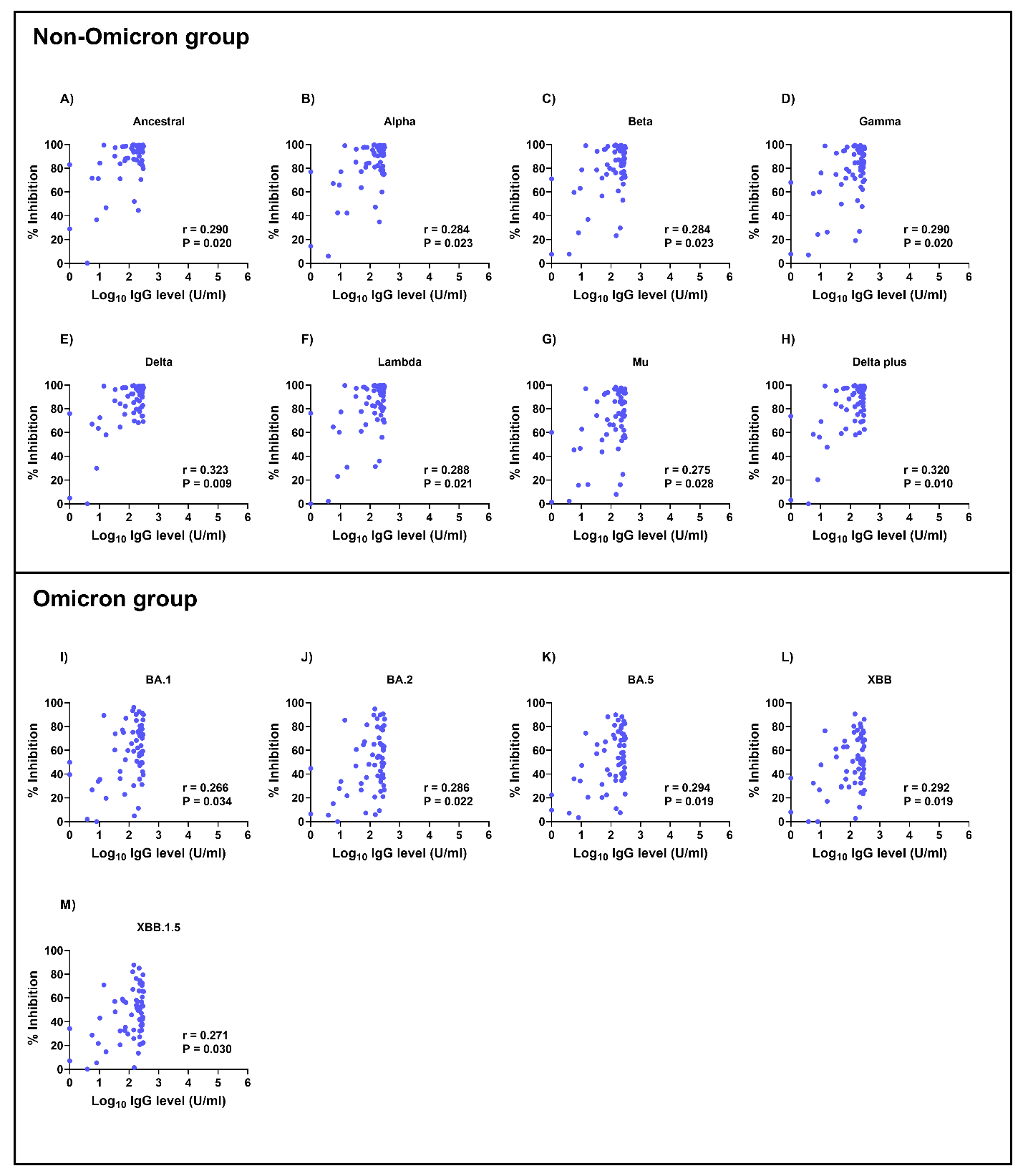


**Supplementary Figure S6** Correlation between RBD-ACE2 blocking and anti-spike IgG antibody levels on day 14 in COVID-19 patients. Blocking antibodies were determined using the sVNT assays (30) (Poolchanuan et al., submitted for publication), and IgG antibodies against the RBD of the SARS-CoV-2 delta variant were determined using ELISA (35). The pairwise correlation coefficient (*r*) was determined using Spearman’s rank correlation.
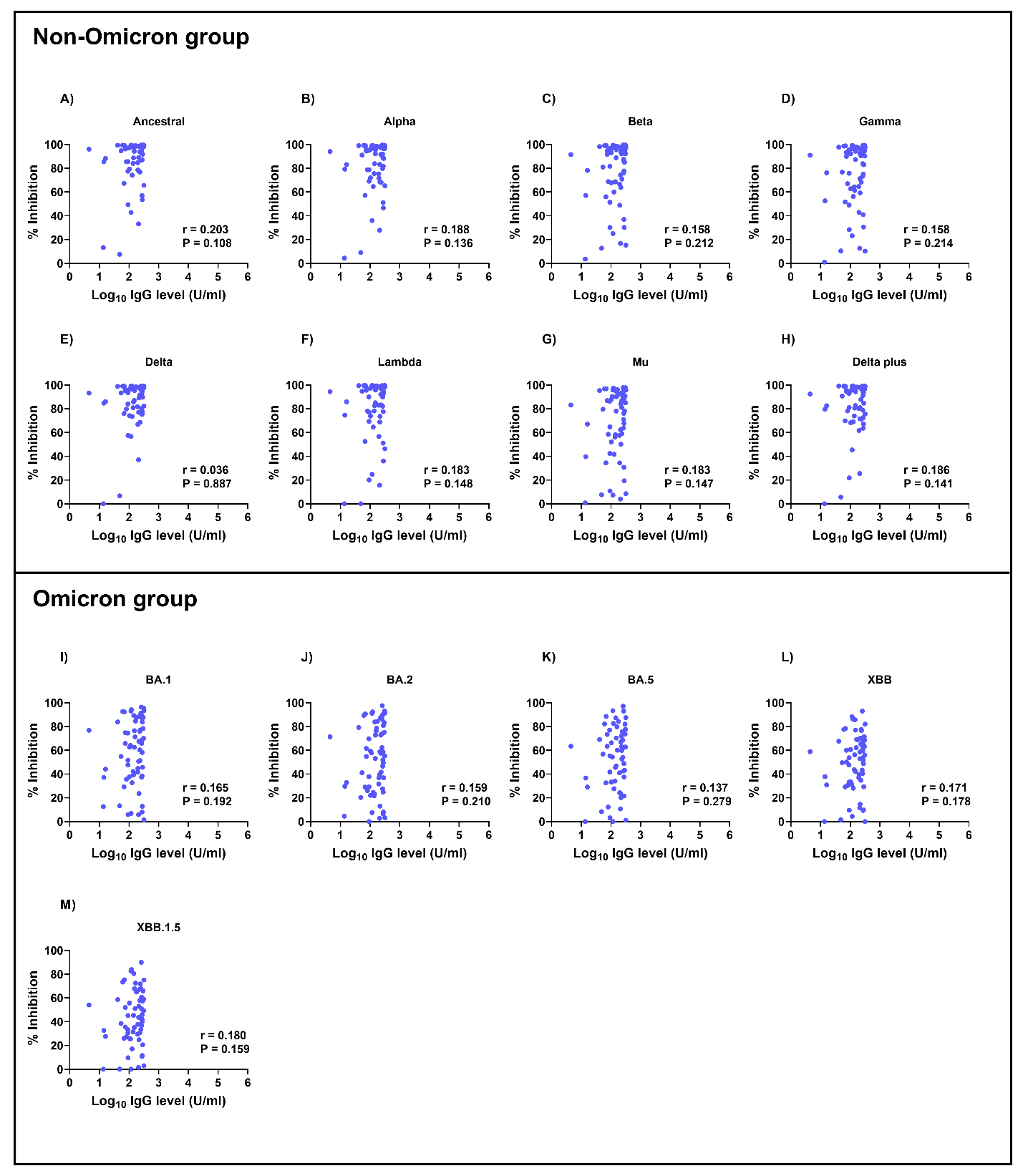


**Supplementary Figure S7** Correlation between RBD-ACE2 blocking and anti-spike IgG antibody levels on day 28 in COVID-19 patients. Blocking antibodies were determined using the sVNT assays (30) (Poolchanuan et al., submitted for publication), and IgG antibodies against the RBD of the SARS-CoV-2 delta variant were determined using ELISA (35). The pairwise correlation coefficient (*r*) was determined using Spearman’s rank correlation.

**Supplementary Table S1** RBD-ACE2 blocking antibody levels against SARS-CoV-2 variants in CoronaVac vaccinated individuals and COVID-19 patients.

| SARS-CoV-2 strains | Median (IQR) percent inhibition of nAbs against SARS-CoV-2 (%) | | | | | |
| --- | --- | --- | --- | --- | --- | --- |
|  | CoronaVac-vaccinated individuals | | | COVID-19 patients | | |
|  | Day 0 | Day 14 | Day 28 | Day 0 | Day 14 | Day 28 |
| Ancestral | 5.1 (1.1-8.6) | 27.5 (20.4-39.5) | 19.4 (13.0-29.8) | 43.1 (8.3-87.0) | 94.9 (84.2-98.3) | 96.1 (81.8-98.8) |
| Alpha | 5.7 (2.3-8.9) | 24.6 (17.8-32.9) | 17.8 (12.2-26.3) | 39.7 (8.7-82.7) | 91.4 (77.8-97.6) | 94.1 (73.7-98.1) |
| Beta | 5.8 (2.2-8.5) | 15.0 (8.2-24.8) | 12.1 (6.2-19.3) | 23.5 (3.1-70.0) | 86.8 (72.6-95.9) | 91.6 (67.2-97.1) |
| Gamma | 6.4 (1.3-8.4) | 12.0 (5.3-23.2) | 7.2 (3.8-15.9) | 22.7 (4.8-65.8) | 84.3 (69.0-94.7) | 90.4 (62.1-96.4) |
| Delta | 5.8 (0-10.6) | 21.1 (12.2-30.7) | 17.0 (9.5-24.8) | 35.6 (4.5-84.1) | 92.7 (77.1-97.5) | 94.0 (80.1-98.2) |
| Lambda | 7.3 (0.5-10.8) | 15.4 (9.0-25.8) | 14.9 (8.7-20.6) | 26.8 (2.2-78.6) | 90.9 (75.4-97.9) | 94.4 (74.3-98.8) |
| Mu | 4.2 (0.4-7.1) | 9.0 (4.4-16.2) | 5.6 (1.6-10.1) | 15.7 (0.2-56.9) | 75.1 (57.1-92.3) | 83.1 (54.1-91.4) |
| Delta plus | 2.6 (0-7.0) | 12.7 (7.8-24.3) | 9.6 (6.2-16.1) | 27.9 (0-78.5) | 91.3 (74.2-96.9) | 93.1 (74.9-98.1) |
| BA.1 | 5.9 (0-10.7) | 6.9 (3.5-13.0) | 4.6 (1.4-10.4) | 11.7 (0-37.7) | 59.9 (42.2-75.5) | 62.6 (38.1-83.9) |
| BA.2 | 3.7 (0-7.1) | 4.2 (1.2-9.0) | 1.7 (0-5.4) | 7.9 (0-25.9) | 49.7 (32.8-68.7) | 54.8 (26.8-78.9) |
| BA.5 | 6.7 (0-14.3) | 9.3 (2.8-14.8) | 0 (0-7.2) | 10.3 (0-30.4) | 53.6 (36.9-70.2) | 55.4 (33.0-74.3) |
| XBB | 2.9 (0-6.5) | 5.5 (0.9-10.6) | 0.8 (0-7.2) | 8.1 (0-29.3) | 50.8 (34.1-68.6) | 53.7 (33.4-67.2) |
| XBB.1.5 | 4.5 (0-8.5) | 5.5 (1.2-9.3) | 1.4 (0-7.5) | 7.4 (0-23.3) | 45.8 (32.2-58.6) | 44.5 (27.6-59.4) |

**Supplementary Table S2** Anti-spike antibodies levels against SARS-CoV-2 in CoronaVac- vaccinated individuals and COVID-19 patients.

| Time point | Median (IQR) U/ml of anti-spike antibodies against SARS-CoV-2 | |
| --- | --- | --- |
|  | CoronaVac vaccinated individuals | COVID-19 patients |
| Day 0 | 0.4 (0.4-0.4) | 1486 (98.2-9473) |
| Day 14 | 121.6 (58.3-234.4) | 17777 (8337-35576) |
| Day 28 | 73.3 (51.3-162.8) | 16834 (4762-33329) |

**Supplementary Table S3** Anti-spike IgG antibodies levels against SARS-CoV-2 in CoronaVac vaccinated individuals and COVID-19 patients.

| Time point | Median (IQR) anti-spike IgG antibodies against SARS-CoV-2 (U/ml) | |
| --- | --- | --- |
|  | CoronaVac-vaccinated individuals | COVID-19 patients |
| Day 0 | 0 | 63.0 (6.4-185.5) |
| Day 14 | 71.5 (37.6-127.9) | 206.5 (78.3-260.8) |
| Day 28 | 55.0 (22.5-95.1) | 187.3 (92.5-265) |
